# Pre-mRNA Secondary Structures Influence Exon Recognition

**DOI:** 10.1371/journal.pgen.0030204

**Published:** 2007-11-16

**Authors:** Michael Hiller, Zhaiyi Zhang, Rolf Backofen, Stefan Stamm

**Affiliations:** 1 Bioinformatics Group, Albert-Ludwigs-University Freiburg, Freiburg, Germany; 2 Institute for Biochemistry, University of Erlangen, Erlangen, Germany; 3 Department of Molecular and Cellular Biochemistry, University of Kentucky, Lexington, Kentucky, United States of America; RIKEN Genomic Sciences Center, Japan

## Abstract

The secondary structure of a pre-mRNA influences a number of processing steps including alternative splicing. Since most splicing regulatory proteins bind to single-stranded RNA, the sequestration of RNA into double strands could prevent their binding. Here, we analyzed the secondary structure context of experimentally determined splicing enhancer and silencer motifs in their natural pre-mRNA context. We found that these splicing motifs are significantly more single-stranded than controls. These findings were validated by transfection experiments, where the effect of enhancer or silencer motifs on exon skipping was much more pronounced in single-stranded conformation. We also found that the structural context of predicted splicing motifs is under selection, suggesting a general importance of secondary structures on splicing and adding another level of evolutionary constraints on pre-mRNAs. Our results explain the action of mutations that affect splicing and indicate that the structural context of splicing motifs is part of the mRNA splicing code.

## Author Summary

Almost all human protein-coding genes contain several exons and introns. Prior to translation, introns have to be removed and exons have to be joined, which happens in a processing step called splicing that generates the mature mRNA. For most genes, certain exons can be either included or excluded from the mature mRNA. It is currently not fully understood which signals are needed to accurately recognize the boundaries of exons in the intron-containing primary transcript. As in transcriptional regulation, enhancer and silencer sequence motifs are crucial for the correct recognition of exons. Splicing regulatory proteins identify these motifs in a sequence-specific manner. In general, these proteins bind to single-stranded RNA. Here, we analyzed local secondary structures of primary transcripts and found that known splicing motifs are preferentially located in a single-stranded context. Experimental tests demonstrated that motifs in single-stranded contexts have a stronger effect on splice site selection than those located in double-stranded regions. These results help to understand the action of human mutations that change the splicing pattern and indicate that local pre-mRNA secondary structures influence exon recognition.

## Introduction

RNA molecules can adopt various conformations in solution by base pairings and hydrophobic interactions. For example, transfer RNA (tRNA) adopts a defined secondary and tertiary structure [1], whereas most messenger RNAs (mRNAs) exhibit only local structures [2]. Defined RNA structures can be recognized by other molecules, as exemplified by aminoacyl-tRNA synthetases that contact with the minor groove of the tRNA acceptor stem [3], which illustrates the functional importance of RNA structure. In contrast, most proteins regulating splice site selection recognize single-stranded, not base-paired RNA. For example, PUF (Pumilio/FBF), Zn-binding, KH (hnRNP K homology) domains, and RRMs (RNA recognition domains) bind to 2–10 nucleotides of single-stranded RNA [4]. Frequently, the RNA part that binds to a protein is in a hairpin loop [5,6]. The sequence specificity of binding is achieved by hydrophobic interactions between RNA bases and amino acids on the surface of the protein, which explains why RNA binding proteins can bind with sequence specificity to unstructured RNA [4].

The large majority of human genes is alternatively spliced [7,8], and the expression of more than one transcript from one gene represents an important mechanism to increase the diversity of a transcriptome and proteome from a limited number of genes [9,10]. In higher organisms, the splice sites do not contain all the information that is required for accurate intron recognition [11]. Additional enhancer and silencer signals located in exons and introns (ESE, ESS, ISE, and ISS for short) are essential for the alternative and constitutive splicing [12]. We refer to them collectively as splicing regulatory motifs. These splicing regulatory motifs bind to RNA binding proteins [4] or other RNAs [13]. There is emerging evidence that pre-mRNA secondary structure plays a role in alternative splicing (reviewed in [14]). For example, a deletion in the mouse fibronectin EDA exon leads to a shift of a critical ESE from single- into double-stranded conformation, which causes exon skipping [15]. The skipping of exon 7 of the *SMN2* gene is correlated with the stability of a stem structure that sequesters the donor splice site [16]. The splicing of mutually exclusive exons in the rat *FGFR2* and *Drosophila DSCAM* gene is regulated by conserved secondary structures [17,18]. Moreover, it has been proposed that sequences surrounding alternative exons might form structures that loop out the exon and prevent its recognition [19,20].

Here, we analyze whether the structural context of binding sites for splicing regulatory proteins has a general importance. We first compiled a large set of experimentally verified splicing enhancer and silencer motifs with their natural sequence context. Calculating the probability of a motif to be single-stranded, we found that splicing motifs are located in a structural context that favors their sequence to be more single-stranded than expected from various controls. These results were confirmed by transfection experiments comparing the effect of known splicing motifs in single- and double-stranded conformation. Furthermore, we show that the structural context of predicted splicing regulatory motifs is under selection. Our findings suggest that pre-mRNA secondary structures are an integral part of splice site recognition.

## Results

### Measurement of Single-Strandedness

To investigate the structural context of splicing motifs, we first developed an appropriate measurement of single-strandedness. Since in vivo mRNA secondary structures are mostly unknown [21], we use the standard approach of predicting structures by energy minimization [22]. For measuring single-strandedness, we compute the probability that all bases in the motif are unpaired (denoted as probability unpaired or PU value) using the equilibrium partition function [23]. Higher PU values indicate higher single-strandedness of the motif. As PU values account for all possible structures, they include possible structural fluctuations and circumvent inaccuracies caused by considering only one energetically optimal structure or a limited number of suboptimal structures. PU values allow the direct comparison of the single-strandedness of splicing motifs.

**Figure 1:**
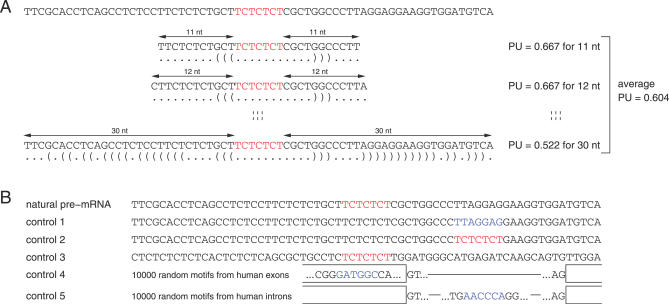
Scheme Illustrating the Computation of PU Values and the Control Datasets (A) The figure shows a silencer site (red) bound by the polypyrimidine tract binding protein in the intron upstream of the mouse c-src exon N1 [53] together with 30 nt of its up- and downstream pre-mRNA sequence context. We computed PU values of the splicing motif for all context lengths from 11 nt up to 30 nt. The optimal secondary structure in dot-parenthesis notation is shown below the sequence. To get a single value that measures the single-strandedness of this motif, we averaged these 20 values. (B) Controls: Given an experimentally verified splicing motif (red) and its natural pre-mRNA context, we randomly selected a substring (blue) with the same length from the flanking regions in control 1. In control 2, we copied the verified motif to a randomly selected position. In control 3, we modified the flanking regions by dinucleotide shuffling [28]. The dataset in controls 4 and 5 consists of 10,000 randomly selected motifs (blue) from human exons (shown as open boxes) and introns (lines), respectively.

In vivo secondary structures of pre-mRNAs are likely to be local rather than global. Local RNA folding is influenced by the length of the flanking sequence context [24], and several lines of evidence indicate that pre-mRNA folding windows are small. First, pre-mRNA is bound by numerous proteins, which influences their ability to fold freely. Second, the formation of secondary structures occurs cotranscriptionally, which favors short-range over long-range base pairing [24]. This model is consistent with the results of kinetic folding algorithms [25]. Third, experiments suggested that pre-mRNA folding is limited to a region of about 50 nt downstream of the transcribing polymerase [26]. For these reasons, we focused on local base pairing and considered all symmetrical context lengths from 11 up to 30 nt up- and downstream of the splicing motif. Thus, for a motif of length 6 nt, we considered sequences with a total length from 28 nt (for context length 11) to length 66 nt (for context length 30). Smaller context lengths were not considered as the resulting sequences rarely form energetically stable structures. We computed the PU value of the splicing motif for all 20 context lengths. To obtain a single PU value for each splicing motif and to reduce a strong dependency on a single fixed context length, we averaged these 20 PU values. Figure 1A illustrates this approach for a binding site of the polypyrimidine tract binding protein.

### Experimentally Determined Splicing Motifs Are Preferentially Located in Single-Stranded Regions

We used the AEdb motif database to obtain an experimentally verified set of high quality splicing regulatory motifs [27]. After filtering (see Materials and Methods), this set comprises 77 exonic and intronic enhancers and silencers with a length up to 9 nt from human, mouse, rat, chicken, *Drosophila*, and several viruses (Table S1). For each motif, we computed the average PU value using the natural pre-mRNA sequence context as described above. To get an overall measure, we averaged these 77 PU values (Table 1; Table S1).

**Table 1:**
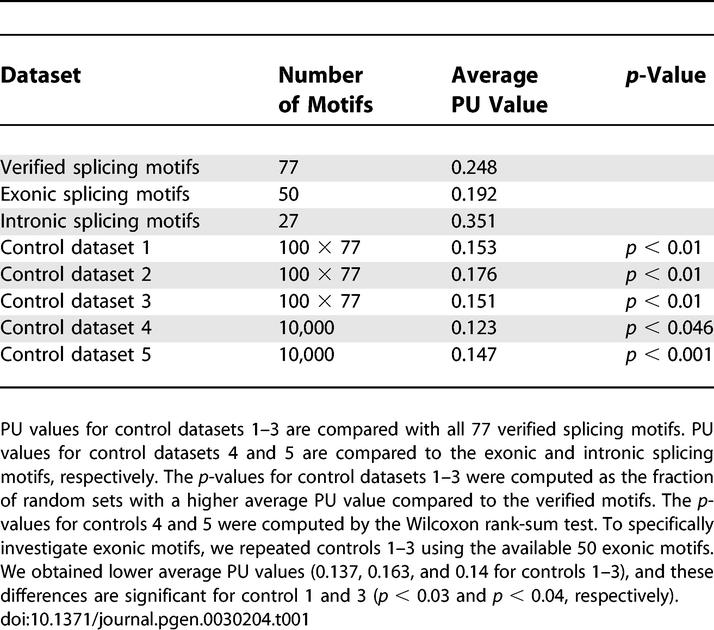
Comparison of Average PU Values for Verified Splicing Motifs and All Control Datasets

To assess whether verified splicing motifs have a preference for single strands, we performed a statistical evaluation controlling for the motif length and the guanine-cytosine content (GC) content that influence the PU values (Figures S1 and S2). First, we randomly chose a new motif of the same length in the up- and downstream flanks of the natural sequence context for all 77 motifs (control dataset 1, Figure 1B). We repeated this 100 times to obtain 100 sets each with 77 randomly chosen motifs. The *p*-value was calculated as the fraction of random sets having a higher average PU value compared to the verified motifs (for example, if all 100 random sets have a lower PU value, the *p*-value is less than 1/100 = 0.01). This control accounts for possible biases in the selection of genes or exons since it uses the same sequences. We observed a substantial drop in the average PU value from 0.25 to 0.15, indicating that verified motifs are significantly more single-stranded (*p* < 0.01, Table 1).

To also account for the sequence bias of the verified motifs, we used another control. We repeated the procedure of control 1 but copied the verified motif to a new position (control dataset 2, Figure 1B). All random sets exhibit a lower single-strandedness (*p* < 0.01). To further verify this effect, we used dinucleotide shuffling [28] to modify the up- and downstream flanks of verified motifs while preserving the motif sequence (control dataset 3, Figure 1B). Again, all 100 randomly generated sets have lower PU values (*p* < 0.01). As a final test, we randomly selected a motif in 10,000 exons and 10,000 introns (control datasets 4 and 5). Since exons and introns have differences in their nucleotide composition [29], we split the 77 motifs into 50 exonic and 27 intronic ones according to their location in the exon-intron structure. As shown in Table 1, we found a significantly higher single-strandedness for exonic and intronic verified motifs compared to exonic and intronic random motifs.

To exclude the possibility that the maximal context length of 30 nt is inappropriate, we repeated the entire analysis with average values for context lengths 11–20 nt as well as 11–50 nt and found consistent results (Table S2). We also tested two other ways to measure the single-strandedness (Text S1; Table S2). It should be noted that even conservative controls with a lower GC content for control datasets 4 and 5 yield a higher single-strandedness for verified motifs (Tables S2 and S3).

The consistent results observed for all tests led us to conclude that naturally occurring, experimentally verified splicing motifs have a significant preference to be single-stranded. Furthermore, the results from controls 2 and 3, which keep the motif sequences, indicate that the higher single-strandedness is attributed to the flanks of the verified motifs rather than the motifs themselves.

### The Activity of Splicing Regulatory Sequences Depends on Their Conformation in the Pre-mRNA

**Figure 2:**
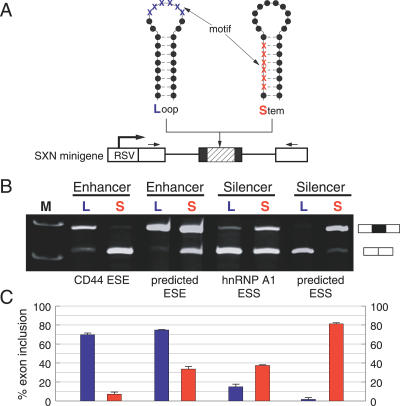
Influence of mRNA Conformation on Splice Site Selection (A) Experimental strategy. The structure of the SXN-derived minigenes is schematically indicated. White boxes represent constitutive globin exons, the black box represents the alternative exon where the motifs are introduced (shaded part). The motifs are either single-stranded in a loop (L) or double-stranded in a stem structure (S). Nucleotides that are part of the motif are indicated by blue (L) and red (S) crosses, flanking nucleotides by black dots. The thick arrow indicates the RSV promoter, small arrows the location of the PCR primers. (B) Analysis of the reporter constructs in vivo. L, motif is in a loop structure (single-stranded); S, motif is in a stem structure (double-stranded). A total of 1 μg of each construct was expressed in HEK293 cells and its RNA was analyzed after 24 h of transfection. An ethidium bromide stained agarose gel of representative experiments is shown. The structure of the products is schematically shown on the right. The sequences containing the motifs (underlined) and their PU values were: CD44 ESE: loop (ATCCATGGGGCTGGATGTGACGTACAACCACAATACGTCACATACTTCCTCTCATGA, PU = 0.998), stem (ATGATGGGTATGTGCGTTGCTTCGGCAACCACAACTCATCGCATACTTCCTCTCATGA, PU < 0.001), predicted ESE: loop (ATCCATGGGGCTGGATGTGACGTAACAAGGCATACGTCACATAGCTTCCTCTCATGA, PU = 0.994), stem (CTACCTTGCGCATGATACGCATGCGCAAGGTAGCACTGCATGAGCTTCCTCACGTTT, PU = 0.164), hnRNP A1 ESS: loop (ATCCATGGGGCTGGATGTGACGTAGTAGGGTATACGTCACATAGCTTCCTCTCATGA, PU = 0.976), stem (CTACCCTACGCATGATACGCATGCGTAGGGTAGCACTGCATGAGCTTCCTCACGTTT, PU = 0.126), predicted ESS: loop (ATCCATGGGGCTGGATGTGACGTAGTAAGTGAATACGTCATATCTTACCTCTCATGA, PU = 0.857), stem (ATCCAGTAAGCTACGCTCCGATGCGTAAGTGAGTCCGCTCACTTACGCATCTCATGA, PU < 0.001). (C) Statistical analysis. The average percent exon inclusion of three independent transfection experiments for S and L constructs is: CD44 ESE, 70% versus 7%; predicted ESE, 75% versus 34%; hnRNP A1 ESS, 15% versus 37%; predicted ESS, 2% versus 81%. Error bars indicate the standard deviation from at least three independent experiments.

Next, we tested the hypothesis that the localization of a splicing regulatory element in a single- or double-stranded RNA structure influences splice site selection in an experimental system. We used the SXN-minigene [30], which has been widely used to analyze the impact of sequences on pre-mRNA processing [31]. This minigene contains an artificial alternative exon between two constitutively spliced globin exons. We inserted splicing enhancer and silencer motifs located either in a single-stranded loop or in a double-stranded stem into this alternative exon (Figure 2A). The single- and double-stranded motifs are located at similar positions in the exon to avoid positional effects [32]. The splicing pattern was analyzed by transfecting the constructs in HEK293 cells followed by detection of the splicing pattern by reverse transcription (RT)-PCR. As enhancer sequences, we used the experimentally well-characterized enhancer of the *CD44* pre-mRNA (CAACCACAA) [33] and a pentamer (CAAGG), which is the core of many computational predicted enhancers [34]. As silencers, we used the experimentally characterized hnRNP A1 (TAGGGT) silencer and a computationally predicted silencer (GTAAGTGA) [35], which was previously experimentally verified in the *HTR2C* pre-mRNA [13].

As shown in Figure 2B and 2C, we observed that the function of these regulatory motifs strongly depends on their localization within an RNA conformation. Enhancers result in stronger exon inclusion when they are located in a loop compared with the location in the stem structure. Likewise, silencers located in a loop lead to stronger exon skipping compared to the loop structure. These data show that the pre-mRNA conformation influences the action of a splicing regulatory element.

### Structural Context of Predicted Splicing Enhancer and Silencer Hexamers

Next, we asked whether we could detect evolutionary selection on the structural context of computationally predicted splicing regulatory motifs. Predicted enhancers, silencers, and “splicing-neutral” hexamers were taken from Stadler et al. [36]. Our strategy to detect differences in single-strandedness of one hexamer was to compare the single-strandedness in enhancer-dependent and silencer-dependent regions. Human exons are dependent on enhancers, while the intronic regions between an authentic and a strong intronic decoy splice site (decoy regions) are dependent on silencers [37]. The single-strandedness of each hexamer was determined as the average PU value for up to 1,000 hexamer occurrences in these datasets. The direct comparison of the PU values of equal hexamers is complicated by a strong negative correlation between the GC content of the hexamer flanking regions and the PU values (*r* = −0.64, *p* < 0.0001), as well as large differences in GC content between exons and decoy regions (Figures S2 and S3). To exclude this GC content bias, we focused only on those cases where selection is strong enough to overcome the overall correlation between PU and GC. Specifically, we considered a hexamer as selected to be single-stranded in exons if, despite more GC rich exonic flanks, the exonic PU value is higher compared to the decoy regions. Likewise, we considered a hexamer as selected to be single-stranded in decoy regions if, despite more GC rich flanks in decoy regions, the PU value is higher compared to exons.

**Figure 3:**
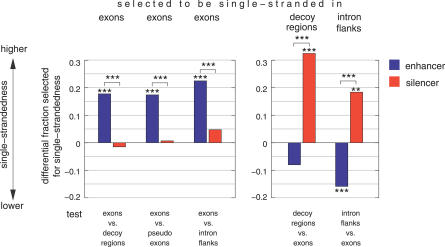
Selection on the Structural Context of Predicted Exonic Enhancer, Silencer, and Splicing-Neutral Motifs The differential fraction of enhancers and silencers that are selected for single-stranded contexts is shown at the *y*-axis. The differential fraction is the difference between the fraction of enhancers/silencers and the fraction of neutral motifs (background); positive values indicate a preference for single-strandedness. To infer selection for single-strandedness, we compared exons with decoy regions, pseudo exons, and intron flanks (*x*-axis). The left side shows the differential fraction of enhancers and silencers that are selected to be single-stranded in exons (more GC rich flanks and higher PU values in exons), the right side the differential fraction in decoy regions and intron flanks (more GC rich flanks and higher PU values in decoy regions and intron flanks). Selection for single-strandedness in pseudo exons cannot be inferred since no enhancer and no silencer motif has more GC rich flanks in pseudo exons. Asterisks above or below bars indicate the significance in a Fisher's exact test comparing enhancers/silencers with neutral motifs; asterisks above brackets indicated the significance comparing enhancers with silencers (***, *p*-value < 0.0001; **: *p* < 0.01).

Using the fraction of splicing-neutral motifs that are selected to be single-stranded as the background fraction, we found that in exons enhancers are significantly more often selected for single-strandedness, while silencers are close to neutral motifs (Figure 3; Table S4). Strikingly, in decoy regions, silencers are significantly more often while enhancers are less often selected to be single-stranded. These results indicate that the high enhancer frequency in exons is further intensified by a tendency for single-strandedness. Likewise, silencers are abundant in decoy regions [35], and in addition, their structural context has a tendency to be selected for single-strandedness. This is further supported by tests with other silencer-dependent regions: pseudo exons (silent intronic regions bounded by strong splice sites [34]) and intronic regions adjacent to authentic splice sites (Figure 3), as well as additional tests that compare enhancer/silencer/neutral motifs having the same number of GCs within and between datasets (Tables S5 and S6). Noteworthy, these differences are more pronounced for exons with weak splice sites and less pronounced for those with strong splice sites, consistent with the idea that the former have an even higher enhancer-dependency (Figure S4). These results indicate a widespread selection pressure on the structural context of splicing motifs.

## Discussion

It is well established that pre-mRNA sequences have enhancing or silencing activities on splice site selection, but the involvement of pre-mRNA secondary structure has not yet been systematically investigated. By analyzing the secondary structure of experimentally verified splicing motifs within their natural pre-mRNA context, we found a higher single-strandedness for these motifs. These results were confirmed by transfection experiments demonstrating that single-stranded splicing motifs exert a stronger effect on the exon usage. Finally, we found that the structural context of predicted splicing motifs is under selection in enhancer- and silencer-dependent regions. Exons, in particular those with weak splice sites, have a higher single-strandedness for enhancers, while silencer-dependent regions have higher single-strandedness for silencers. As most exons are dependent on several splicing regulatory motifs, these findings suggest a general importance of pre-mRNA secondary structures on the splicing outcome. They indicate that secondary structures are a part of the “mRNA splicing code” that determines exon recognition. Selection on secondary structures could also explain the observed constraints on synonymous codon sites [38] and the conservation of large intronic regions adjacent to alternative exons [39]. We propose that a coding exon is subjected to at least three different selection pressures: (i) preserving the coding sequence, (ii) preserving the sequence of splicing motifs, and (iii) preserving an appropriate structural context for these splicing motifs. Selection on the coding sequence is likely to be the strongest pressure. This is consistent with results that splicing motifs are interchangeable [36] and our finding that intronic motifs are more single-stranded than exonic ones (Table 1).

Most splicing regulatory motifs are recognized by sequence-specific RNA binding proteins that make direct contacts with unpaired RNA bases [4]. This provides a mechanistic explanation for the inhibitory role of double strands on RNA–protein and mRNA–microRNA interactions [40,41] and for our observation that splicing regulatory motifs are located in single-stranded regions. In contrast to this general trend, a few individual motifs are located in a somewhat double-stranded conformation (Table S1). It remains to be determined which trans-acting factors bind to these motifs or whether a specific experimental situation influenced the results. It is also possible that proteins binding to the proximity of the double-stranded motifs change their single-strandedness or that RNA helicases unwind them [42].

**Figure 4:**
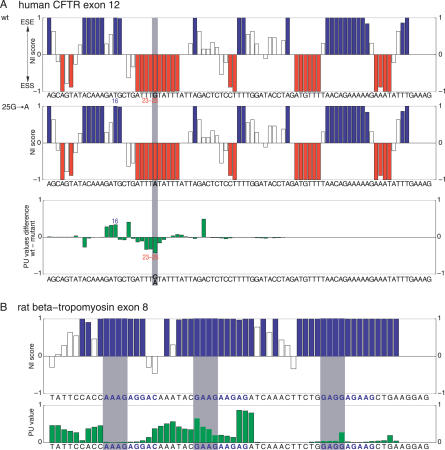
mRNA Secondary Structures Can Explain Experimental Observations (A) A structural change explains exon skipping by the 25G->A mutation in the human *CFTR* exon 12**.** NI scores [36] for the wild-type (wt) and the 25G->A mutation (highlighted gray) indicate the strength of ESE (blue, positive score) and ESS (red, negative score) motifs. Hexamers with an NI score >0.8 and <−0.8 are considered to be strong ESEs and ESSs, respectively; the remaining hexamers are “splicing-neutral” (open boxes). A positive PU value difference (PU_wild-type_ minus PU_mutant_) indicates a higher single-strandedness for the respective hexamer in the wild-type exon and vice versa. Bars give the NI score or the PU value change for the hexamer starting at this position. Compared to the wild-type sequence, the 25G->A mutation does not change ESE or ESS motifs. However, the ESE at position 16 becomes more double-stranded in the 25G->A mutant, and the ESSs at position 23–25 become more single-stranded, explaining the decrease in exon inclusion from 80%–25% [43]. (B) Due to their structural conformation, a predicted ESE is not active in the exon 8 of the rat beta-tropomyosin gene. The three purine-rich ESE candidates with NI scores of 1 characterized in [54] are shown in blue letters. Bars for hexamers that overlap these three investigated 9-mers are highlighted gray. While the first motif is highly double-stranded, the second and the third motif have at least one hexamer in a more single-stranded context. This explains experimental results as the mutation of the second and to a lesser extent of the third motif affects splicing, while mutating the first motif shows no effect [54]. Thus, observed splicing effects correlate with the single-strandedness of these three motifs. Average PU values using the context lengths 11–30 nt are shown in (A) and (B).

Our findings can explain previously observed splicing effects of mutations that do not change splicing regulatory elements. For example, a silent mutation in human *CFTR* exon 12 that reduces exon inclusion from 80%–25% [43] does not create or destroy splicing motifs, but leads to a higher single-strandedness of existing ESSs and a lower single-strandedness of an existing ESE (Figure 4A). Other examples for mutations in human *HPRT1* exon 8 are shown in Figures S5 and S6. Likewise, secondary structures can explain why mutations that change splicing motifs sometimes show no splicing effect. Most likely, the affected motifs are highly double-stranded in these cases; exemplified for motifs in rat beta-tropomyosin exon 8 (Figure 4B), human *MAPT* alternative exon 10 (Figure S7), and human *HPRT1* exon 8 (Figures S8 and S9). Thus, the structural context should be taken into account in mutagenesis experiments [44], where observed splicing effects are usually interpreted as changes in regulatory motifs. To facilitate such analyses, we provide a web resource (http://biwww2.informatik.uni-freiburg.de/Software/NIPU) that allows a comparison between PU values and ESE/ESS scores [36]. As the formation of secondary structures depends on the speed of transcription [24], the effect of the promoter on splicing [45] might be partially explained by differential structure formation. Furthermore, secondary structures provide useful information in sequence motif finding [46] and will improve the computational detection of splicing motifs [31,34,47] and gene prediction algorithms [35].

## Materials and Methods

### Measurement of single-strandedness.

PU values were computed as described in [46]. Briefly, the PU value for the region *a* to *b* in an mRNA sequence is defined as

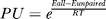
where *E_all_* is the free energy of the ensemble of all structures, *E_unpaired_* is the free energy of the ensemble of all structures that have the complete region *a* to *b* unpaired, *R* is the universal gas constant, and *T* is the temperature. We computed *E_all_* and *E_unpaired_* using the partition function version of RNAfold [48]. For *E_unpaired_,* we assure that the region *a* to *b* is unpaired by applying additional constraints (RNAfold parameter-C). Other measurements of single-strandedness are described in the Text S1.

### Experimentally determined splicing motifs.

We carefully examined the literature for all splicing motifs listed in the motif database of AEDB (http://www.ebi.ac.uk/asd/aedb/) [27]. We checked the consistency of the listed genes, species, and motif sequences using the respective publications. Only motifs that were demonstrated to influence splicing in their natural context were considered. We excluded motifs whose annotation was based solely on computational predictions, cases where the exact motif location is not given in the publication, or where we could not find the given motif in the respective gene. Motifs that presumably act by forming secondary structures instead of representing a protein binding site and motifs that were predicted on the basis of the splicing effect of a SNP were also excluded. Since three-dimensional structures of single-stranded RNA binding proteins indicate that they usually contact only a few residues [4], we discarded all motifs with a length of more than 9 nt as these longer motifs are likely to contain a core binding site at a location that was not experimentally determined. We removed redundancy by requiring that each motif has a unique location within a gene.

### Control datasets.

For control datasets 1–3, we randomly generated 100 motif sets each with 77 random motifs by repeating the following procedures 100 times for each verified motif. Control dataset 1: We randomly selected a new position within the 150-nt up- and downstream flanking regions of the verified motif. The subsequence starting at this position with the same length as the verified motif was taken as the new motif. Control dataset 2: We used the same procedure as for control 1 but replaced the subsequence starting at the new position by the verified motif. Control dataset 3: Dinucleotide shuffling was done with Dishuffle [28] separately for the 300-nt up- and downstream flanks of the motif. The Dicodonshuffle program, which shuffles dicodons while preserving the coding sequence, could not be used because the output was not variable enough to yield 100 different sets. It should be noted that all control sequences were repeatedly derived from the same set of verified sequences. They are therefore not necessarily independent and the Wilcoxon rank-sum test cannot be used. To evaluate them statistically, we calculated the *p*-value as the fraction of random sets having a higher average PU value compared to the verified motifs. Control datasets 4 and 5: We downloaded from the University of California Santa Cruz Genome Browser the Human genome assembly (hg17, May 2004) as well as RefSeq transcript annotations (refGene.txt.gz, January 2005). Then, we extracted all internal exons and all 300-nt flanks from all introns. Redundancy was removed by using RSA-tools (http://rsat.ulb.ac.be/rsat/purge-sequence_form.cgi) with a minimal match length of 50 nt and at most three mismatches. We randomly selected 10,000 motifs within the exons (introns) while keeping the length distribution of the exonic (intronic) splicing motifs. All intronic motifs are at least 50 nt away from the splice site. The Wilcoxon rank-sum test was used to test whether verified and random motifs come from the same distribution. In all controls, we used the length distribution of the verified splicing motifs and controlled for the GC content (Table S3). Datasets with a lower and higher GC content for control 4 and 5 were generated by randomly discarding entries with a GC content of smaller and greater than 0.5. Statistical tests (Fisher's exact test, Wilcoxon rank-sum test) were performed using R (http://www.r-project.org/).

### Minigene experiments.

Splicing assays and cotransfection were performed as described [49], employing the SXN derivatives. Briefly, 1 μg of indicated minigenes were transfected into HEK293 cells using the calcium phosphate method. RNA was isolated 24 h post-transfection by using the total RNA kit (Qiagen). Reverse transcription was performed in a total volume of 8.5 μl by using 2 μl RNA, 1 μl oligo (dT) (0.5 mg/ml), 1 μl first-strand buffer (Invitrogen), 10 mM DTT, 1 mM dNTP, 5 units SuperScript II RT (Invitrogen) at 42 °C for 60 min. To ensure that only plasmid-derived minigene transcripts were detected, subsequent amplification was performed using vector-specific primers (fwd-CCATTTGACCATTCACCACA, rev-CACTCCTGATGCTGTTATGG). RT-PCR products were resolved on ethidium bromide-stained agarose gels. The ratio of exon inclusion to exon skipping was determined using the Image G program. To detect possible unspliced products, RNA samples were treated with DNAse and after reverse transcription, unspliced RNA was amplified using 4′ extension times. Using these conditions, no unspliced RNA could be observed.

### Predicted splicing motifs.

The exon set corresponds to the human exons used for control dataset 4. The definition of pseudo exons was adopted from [34] as intronic region with a length between 50 and 250 nt, flanked by two splice sites having a score of at least 8.5. We used a maximum entropy-based model for the quantification of the splice site strength as described in [50], which we computed using the Web server http://genes.mit.edu/burgelab/maxent/Xmaxentscan_scoreseq.html. All pseudo exons that overlap with spliced ESTs were excluded. To search for “decoy regions,” we scored all potential splice sites in the 100-nt intron flanks for introns longer than 400 nt using the maximum entropy model [37]. A decoy donor region is the region between a real donor site and a downstream decoy donor site (located at least 6 nt downstream), which has a higher score. A decoy acceptor region is the region between a real acceptor site and an upstream decoy acceptor site (located at least 15 nt upstream), which has a higher score. Decoy donor and acceptor regions were merged. The intron flank dataset consists of the regions +10...+70 from a donor site and −80... −20 from the acceptor site of the human exons. Redundancy in each dataset was removed using RSA-tools with a minimal match length of 30 nt and at most two mismatches.

For each dataset, we randomly selected up to 1,000 occurrences for each of the 4,096 hexamers. Then, we determined the single-strandedness for one hexamer by averaging the PU values (using context lengths 11–30 nt) for each of the hexamer occurrences. The average GC content of one motif was computed for the 66-nt region (30 nt up/downstream context + 6 nt of the motif). The 4,096 hexamers were divided into exonic enhancers (neighborhood inference (NI) score > 0.8), silencers (NI score < −0.8), and splicing-neutral motifs (−0.8 ≥ NI score ≤ 0.8), as suggested in Stadler et al. [36].

## Supporting Information

Figure S1 Influence of the Motif Length on PU Value We selected 10,000 random motifs with lengths 3–9 nt from a large set of human exons and introns and computed PU values (context lengths 11–30 nt). As shown in this figure, PU values strongly depend on the motif length (correlation coefficient *r* = −0.967 for exons, *r* = −0.972 for introns, *p* = 0.0004). The correlation coefficient for the 77 verified motifs is *r* = −0.803.
(22 KB PDF)

Figure S2 Correlation between PU Values and the GC Content The GC content influences the stability of secondary structures as C-G base pairs are more stable than A-U pairs. Therefore, GC rich regions tend to have lower PU values. For human exons, we randomly selected up to 1,000 occurrences for each of the 4,096 hexamers. Then, we determined the single-strandedness for one hexamer by averaging the PU values (using context lengths 11–30 nt) for each of the hexamer occurrences. The average GC content of one motif was computed for the 66-nt region (30 nt up/downstream context + 6 nt of the motif). The average PU value and the average GC content are significantly negatively correlated (*r* = −0.644, *p* < 0.0001).
(78 KB PDF)

Figure S3 Boxplots for the GC Content of the Different Datasets Boxplots show the different GC content distribution for all 4,096 hexamers in the datasets (*x*-axis). The GC content (for the 30 nt up/downstream context + 6 nt of the motif) for one motif was averaged over the up to 1,000 hexamer occurrences in one dataset. Exons have a higher GC content than intronic regions. In particular, pseudo exons are AT rich.
(14 KB PDF)

Figure S4 Selection on the Structural Context of Predicted Exonic Enhancers, Silencers, and Neutral Motifs Comparing Weak and Strong Exons with Decoy Regions, Pseudo Exons, and Intron Flanks We collected a set of human exons with weak and strong splice sites. Weak donor and acceptor sites have maximum entropy scores [50] lower than 7 and 6.5, respectively. Strong donor and acceptor sites have scores higher than 10 and 10.5, respectively. These thresholds correspond to the first and third quantile of the score distribution. The dataset of strong (weak) exons comprises up to 1,000 occurrences for each hexamer from the exonic regions +1 to +50 downstream of a strong (weak) acceptor and −50 to −1 upstream of a strong (weak) donor. Then, we compared weak and strong exons with decoy regions, pseudo exons, and intron flanks. The differential fraction (difference between the fraction for enhancers/silencers and neutral motifs) of enhancers and silencers that are selected for single-stranded contexts is shown at the *y*-axis. The comparisons to infer selection for single-strandedness are given on the *x*-axis. Selection for single-strandedness in pseudo exons cannot be inferred since no enhancer and no silencer motif has more GC rich flanks in pseudo exons. Asterisks above or below enhancer or silencer bars indicate the significance in a Fisher's exact test compared to neutral motifs; asterisks above brackets indicated the significance comparing enhancers with silencers (***, *p* < 0.0001; **, *p* < 0.001; *, *p* < 0.05). Horizontal dotted lines indicate the differential fractions of human exons (taken from Figure 3). These tests show that enhancers have an even higher tendency to be selected for single-strandedness in weak exons.
(21 KB PDF)

Figure S5 A Structural Change and the Structural Context of Affected Splicing Motifs Explain Skipping of Human *HPRT1* Exon 8 by the 589G->T, 592T->A Double-Mutation ESE/ESS scores and PU values for the wild-type (wt) exon 8 and the 589G->T, 592T->A double-mutant are shown. The positions of the mutations are highlighted gray. Neighborhood inference (NI) scores [36] indicate the strength of ESE (blue, positive score) and ESS (red, negative score) motifs (NI scores >0.8 and <−0.8 indicate strong ESEs and ESSs, respectively). Bars give the NI score or the PU value for the hexamer starting at this position.
The mutations lead to the loss of one ESE (position 54) and one ESS (position 59) compared to the wild-type exon; both motifs are somewhat double-stranded in the wild-type exon (PU ≈ 0.2 and 0.1, respectively). Compared to the wild-type exon, the mutant has two new ESE motifs (positions 58 and 60) and two new ESS motifs (positions 55 and 56). However, both new ESSs are highly single-stranded (PU ≈ 0.7), while the only one of the new ESEs is single-stranded (position 58, PU ≈ 0.7; position 60, PU < 0.1). In addition, the unchanged ESS motifs at position 51–53 become much more single-stranded in the mutant (PU values increase from ∼0.2 to ∼0.6). Thus, the change in splicing motifs (equal number of created and destroyed ESEs/ESSs) can probably not fully explain why 50% of the mutant exon 8 is skipped [51]. However, structural changes provide an additional explanation for the splicing effect of the mutations. It is also interesting to note that the strong ESS block upstream (AAGTTTGTT) is highly double-stranded in the wild-type exon. This analysis was done with the Web resource http://www.bioinf.uni-freiburg.de/Software/NIPU/.
(60 KB PDF)

Figure S6 A Structural Change Can Explain Skipping of Human *HPRT1* Exon 8 by the 550delC Mutation ESE/ESS scores and PU values for the wild-type (wt) exon 8 and the 550delC mutant (highlighted gray) are shown. See the legend of Figure S5 for description of NI scores and PU values. The 550delC mutant leads to a new ESE at position 15, thus it is unexpected that the mutant exon is completely skipped [51], since neither existing ESEs are destroyed nor new ESSs are created. Considering secondary structures, we note that the new ESE is highly double-stranded (PU < 0.1), thus is not likely to contribute to exon inclusion. Furthermore, the existing ESE at position 10 (as well as the weaker ESEs at 9 and 11) looses much of its single-strandedness (PU value drop from ∼0.95 to ∼0.35). Assuming that this ESE is crucial for exon inclusion, the shift to a more double-stranded conformation would explain skipping of the mutant exon.
(53 KB PDF)

Figure S7 Changes in Double-Stranded Splicing Motifs Do Not Affect Splicing of Human *MAPT* Exon 10 ESE/ESS scores and PU values for the 3′ part of the wild-type (wt) exon 10 and the P301L (A) and P301S (B) mutant are shown. The positions of the mutations are highlighted gray. See the legend of Figure S5 for description of NI scores and PU values. The P301L mutation (codon CCG->CTG) creates two new ESSs (positions 16 and 18), thus it is expected to reduce exon 10 inclusion. Similarly, the P301S mutation (codon CCG->TCG) destroys two strong ESEs (positions 15 and 17) and one weaker ESE (position 16). However, both mutations do not affect the inclusion of exon 10 [52]. This is likely explained by the secondary structure context of the created or destroyed motifs that is highly double-stranded in the wild type and the mutants (PU values < 0.1).
(49 KB PDF)

Figure S8 Changes in Double-Stranded Splicing Motifs Do Not Affect Splicing of Human *HPRT1* Exon 8 ESE/ESS scores and PU values for the wild-type (wt) exon 8 and the 601G->T mutant (highlighted gray) are shown. See the legend of Figure S5 for description of NI scores and PU values. The mutation results in the loss of an ESE (position 65) and the creation of four ESSs (positions 66–69). Thus, the mutant exon is expected to be skipped to some extent. However, no change in exon inclusion was described for this mutation [51]. This can be explained by the secondary structure context of the affected motifs, as the destroyed ESE is highly double-stranded in the wt exon (PU < 0.05), and the new ESSs are double-stranded in the mutant exon (PU < 0.05).
(58 KB PDF)

Figure S9 Changes in Double-Stranded Splicing Motifs Do Not Affect Splicing of Human *HPRT1* Exon 8 ESE/ESS scores and PU values for the wild-type (wt) exon 8 and the 562G->T mutant (highlighted gray) are shown. See the legend of Figure S5 for description of NI scores and PU values. The mutation strengthens two ESSs (positions 29 and 30) but was not described to lead to exon skipping [51]. This can be explained by the fact that these two ESSs are highly double-stranded (PU < 0.1).
(53 KB PDF)

Table S1 General Information, GC Content, and Measurements of Single-Strandedness for the 77 Experimentally Determined Splicing Motifs PU20 means average PU values for context lengths 11–20 nt. PU30 means average PU values for context lengths 11–30 nt. PU50 means average PU values for context lengths 11–50 nt. This notation is also used for EF and ED.
(51 KB PDF)

Table S2 PU, EF, ED values, and *p*-Values for Verified Splicing Motifs and All Controls The *p*-values for controls 1–3 were computed by counting the number of test sets with a higher average single-strandedness compared to the all 77 verified motifs. The *p*-values for controls 4 and 5 were computed by the Wilcoxon rank-sum test since the data are not normally distributed (controls 4 and 5 were compared to exonic and intronic splicing motifs, respectively). *p*-Values in italics are not significant at the 0.05 level. We tested several controls with the notion that the likelihood of a real effect increases if all controls show a significant difference. As PU values depend on the length of the motif (Figure S1), we used the length distribution of the verified splicing motifs in all controls. Furthermore, we have to exclude that any observed differences in single-strandedness are attributed to differences in the GC content. For controls 4 and 5, we additionally selected exonic and intronic motif occurrences yielding a lower, equal, and higher average GC content. Consistently, all PU, EF, and ED values for all context lengths and all controls show a lower single-strandedness compared to the verified motifs, even for the conservative controls with a lower GC content. Thus, differences in the GC content cannot explain the observation that verified splicing motifs are more single-stranded. The observed effect is further supported by different measurements of single-strandedness (EF and ED) and different context lengths (11–20 nt and 11–50 nt).
(52 KB PDF)

Table S3 GC Content for the Verified Motif Datasets and All Controls The GC content for control dataset 1–3 was computed by averaging over the 100 sets. GC content values in bold are higher than the values for the verified motifs.
(44 KB PDF)

Table S4 Number of ESEs, ESSs, and Neutral Motifs That Are Selected for Single-Strandedness Comparing Exons with Decoy Regions, Pseudo Exons, and Intron Flanks The differential fractions and the *p*-values are presented in Figure 3.
(48 KB PDF)

Table S5 Comparison of PU Values of Splicing Motifs between Datasets For this test, we call a hexamer “winner” if it has a higher PU value in >50% of all comparisons to other hexamers with the same number of GCs in the hexamer. Here, we focus only on the GC content of the motif, since the number of GCs in the hexamer is nearly perfectly correlated with the average GC content of the up- and downstream 30-nt flanks (*r* = 0.94). Therefore, this procedure also accounts for the GC content. We compared the PU values of ESEs in exons with the PU values of ESEs in pseudo exons. For example, we compared the exonic PU value of the ESE ACCATT (PU = 0.2186, number of GCs = 2) with the PU value in pseudo exons for the ESE CAGAAA (PU = 0.1982), AGATGT (PU = 0.1158), TAAACC (PU = 0.3455), etc. and determined the percentage of cases where the exonic PU value is higher. A total of 478 of the 979 (48.8%) ESEs are winners (have a higher PU in >50% of the comparisons). Comparing ESSs in exons with ESSs in pseudo exons shows that only 194 of the 496 ESSs (39.1%) are winners, which is significantly lower than the percentage of ESE winners. Thus, ESEs have a 9.7% (48.8%–39.1%) higher chance to be a winner. We also calculated the number of winners for splicing neutral motifs. Compared to neutral motifs, ESEs have a 6.1% (48.8%–42.7%) higher chance, while ESSs have a 3.6% lower chance (39.1%–42.7%) to be a winner. The comparison of exons versus decoy regions and intron flanks are consistent with the results for pseudo exons. ^a^Number of ESE (ESS, neutral) hexamers that are winners when comparing exons with decoy regions, pseudo exons, and intron flanks; ^b^Total number of ESEs, ESSs, and splicing neutral motifs.
(38 KB PDF)

Table S6 Comparison of PU Values of Splicing Motifs within Datasets We call a hexamer “winner” if it has a higher PU value in >50% of all comparisons to other hexamers with the same number of GCs in the hexamer. We compared the PU values of ESEs with the PU values of splicing neutral motifs within one dataset. A total of 56.8% of the ESEs are winners in exons, but significantly fewer ESEs (50.5%) are winners in pseudo exons, which indicates that ESEs have a higher chance to be a winner in exons (difference 6.3%). Comparing the PU values of ESSs with the PU values of splicing neutral motifs within one dataset shows that 21.8% of the ESSs are winners in exons but 31.5% are winners in pseudo exons, which indicates that ESSs have a significantly lower chance to be a winner in exons (difference −9.7%). Comparing exons with decoy regions and intron flanks leads to consistent results. ^a^Number of winners comparing ESEs with neutral and ESSs with neutral motifs within one dataset;^ b^Total number of ESEs or ESSs.
(30 KB PDF)

Text S1 Additional Measurements of Single-Strandedness (50 KB PDF)

## Abbreviations

GC: guanine-cytosine content
mRNA: messenger RNA
NI: neighborhood inference
PU: probability unpaired
