# Supplementary material for: Pre-mRNA Secondary Structures Influence Exon Recognition
Source: PLoS Genet. 2007 Nov 16;3(11):e204. doi: 10.1371/journal.pgen.0030204 (PMC2077896; doi:10.1371/journal.pgen.0030204)
Supplement: Supplementary file 1 [file pgen.0030204.sd001.pdf]

Supporting Text

Additional measurements of single-strandedness:

In contrast to the structure of a protein, RNAs often have more flexible and dynamic structures, which hamper the experimental structure determination. Therefore, the secondary structure (the set of all base pairings) is usually predicted by energy minimization (1). Since the natural occurring secondary structure is not always the structure with the minimal free energy, a set of suboptimal structures is often considered. Structures with a similar free energy can differ greatly and a single RNA sequence was shown to adopt more than one structure and to perform more than one function (2). However, up to which energy threshold suboptimal structures are considered is an arbitrary choice. These problems can be circumvented by computing the equilibrium partition function and the base pair probabilities (3). Base pair probabilities represent the likelihood that two bases form a base pair in the structural ensemble.

In addition to the PU value (see Methods), we also measure the single-strandedness of a motif by: (i) the expected fraction of bases in the motif that are unpaired (denoted as EF value) (4) and (ii) the energy difference between the ensemble of all structures and the ensemble of the structures that do not have base pairs in the motif (denoted as ED value). EF is defined as

$$EF = 1 - \frac{\sum_{i=a}^{b} \sum_{j=1}^{N} p_{i,j}}{b-a+1}$$

where N is the length of the sequence, a and b indicate the start and end of the motif, and $p_{i,j}$ is the probability that base i and j are paired. We used the RNAfold program with default parameters (5) to compute the base pair probability matrix of a given sequence. ED is defined as $ED = E_{all} - E_{unpaired}$, where E_{all} is the free energy of the ensemble of all structures and $E_{unpaired}$ is the free energy of the ensemble of all structures that have the complete motif unpaired. $E_{unpaired}$ can be computed with the constraint function of RNAfold (parameters -p0 -C) (5).

Higher values for EF indicate a higher single-strandedness of the motif. In contrast, the higher ED the more stable structures have at least one base pair in the motif, which contributes to a higher energy difference between the two ensembles. Thus, lower ED values indicate a higher single-strandedness.

1. Zuker, M. and Stiegler, P. (1981) Optimal computer folding of large RNA sequences using thermodynamics and auxiliary information. *Nucleic Acids Res*, **9**, 133-148.
2. Schultes, E.A. and Bartel, D.P. (2000) One sequence, two ribozymes: implications for the emergence of new ribozyme folds. *Science*, **289**, 448-452.
3. McCaskill, J.S. (1990) The equilibrium partition function and base pair binding probabilities for RNA secondary structure. *Biopolymers*, **29**, 1105-1119.
4. Hiller, M., Pudimat, R., Busch, A. and Backofen, R. (2006) Using RNA secondary structures to guide sequence motif finding towards single-stranded regions. *Nucleic Acids Res*, 34, e117.
5. Hofacker, I.L. (2003) Vienna RNA secondary structure server. *Nucleic Acids Res*, 31, 3429-3431.
