# Supplementary material for: Pre-mRNA Secondary Structures Influence Exon Recognition
Source: PLoS Genet. 2007 Nov 16;3(11):e204. doi: 10.1371/journal.pgen.0030204 (PMC2077896; doi:10.1371/journal.pgen.0030204)
Supplement: Supplementary file 2 [file pgen.0030204.sg001.pdf]

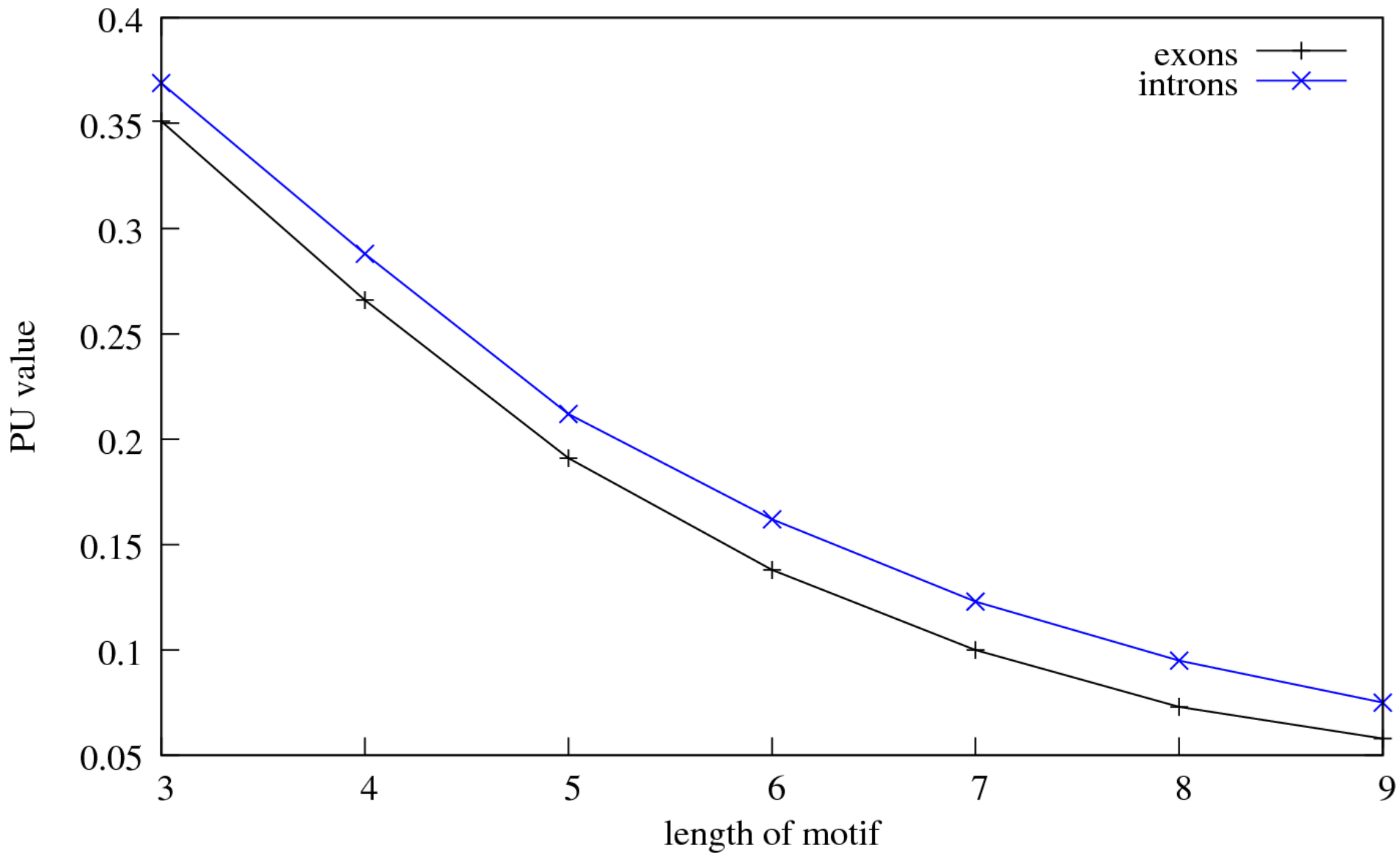

PU value
length of motif
exons
introns
0.4
0.35
0.3
0.25
0.2
0.15
0.1
0.05
3
4
5
6
7
8
9
