# Supplementary material for: Pre-mRNA Secondary Structures Influence Exon Recognition
Source: PLoS Genet. 2007 Nov 16;3(11):e204. doi: 10.1371/journal.pgen.0030204 (PMC2077896; doi:10.1371/journal.pgen.0030204)
Supplement: Supplementary file 3 [file pgen.0030204.sg002.pdf]

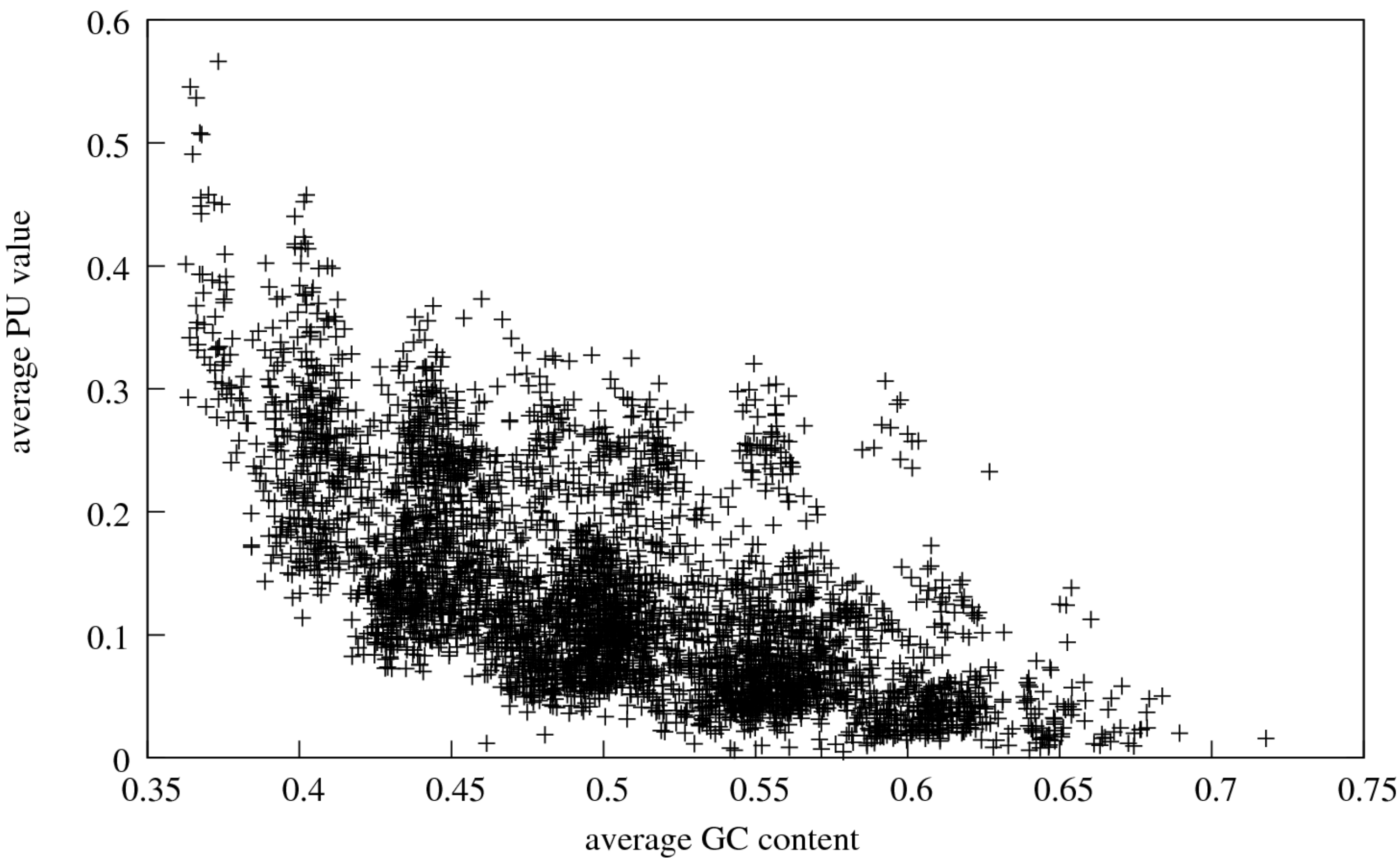
average PU value
0.6
0.5
0.4
0.3
0.2
0.1
0
0.35
0.4
0.45
0.5
0.55
0.6
0.65
0.7
0.75
average GC content
