# Supplementary material for: Pre-mRNA Secondary Structures Influence Exon Recognition
Source: PLoS Genet. 2007 Nov 16;3(11):e204. doi: 10.1371/journal.pgen.0030204 (PMC2077896; doi:10.1371/journal.pgen.0030204)
Supplement: Supplementary file 4 [file pgen.0030204.sg003.pdf]

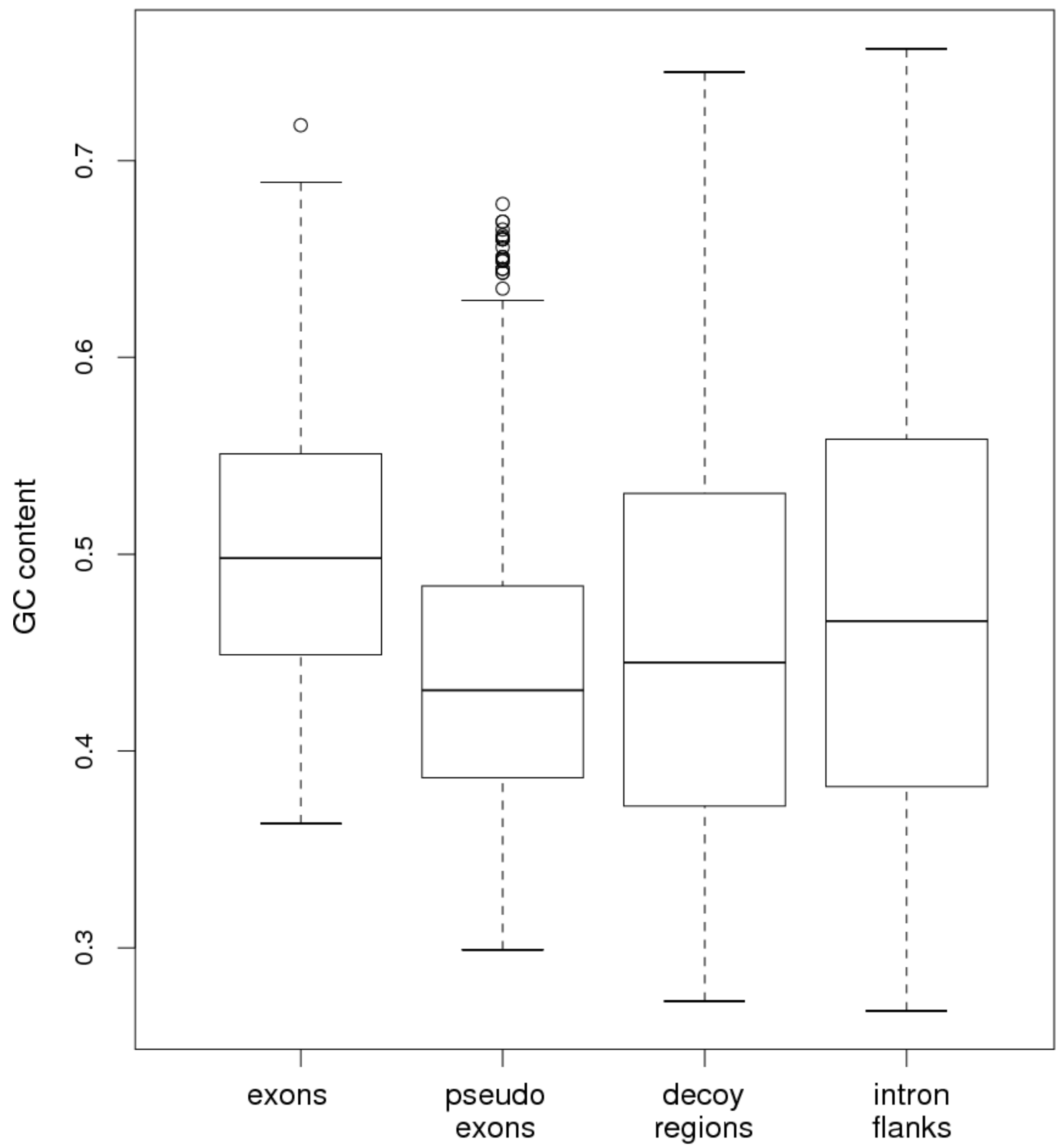
GC content
0.3
0.4
0.5
0.6
0.7
exons
pseudo exons
decoy regions
intron flanks
