# Supplementary material for: Pre-mRNA Secondary Structures Influence Exon Recognition
Source: PLoS Genet. 2007 Nov 16;3(11):e204. doi: 10.1371/journal.pgen.0030204 (PMC2077896; doi:10.1371/journal.pgen.0030204)
Supplement: Supplementary file 5 [file pgen.0030204.sg004.pdf]

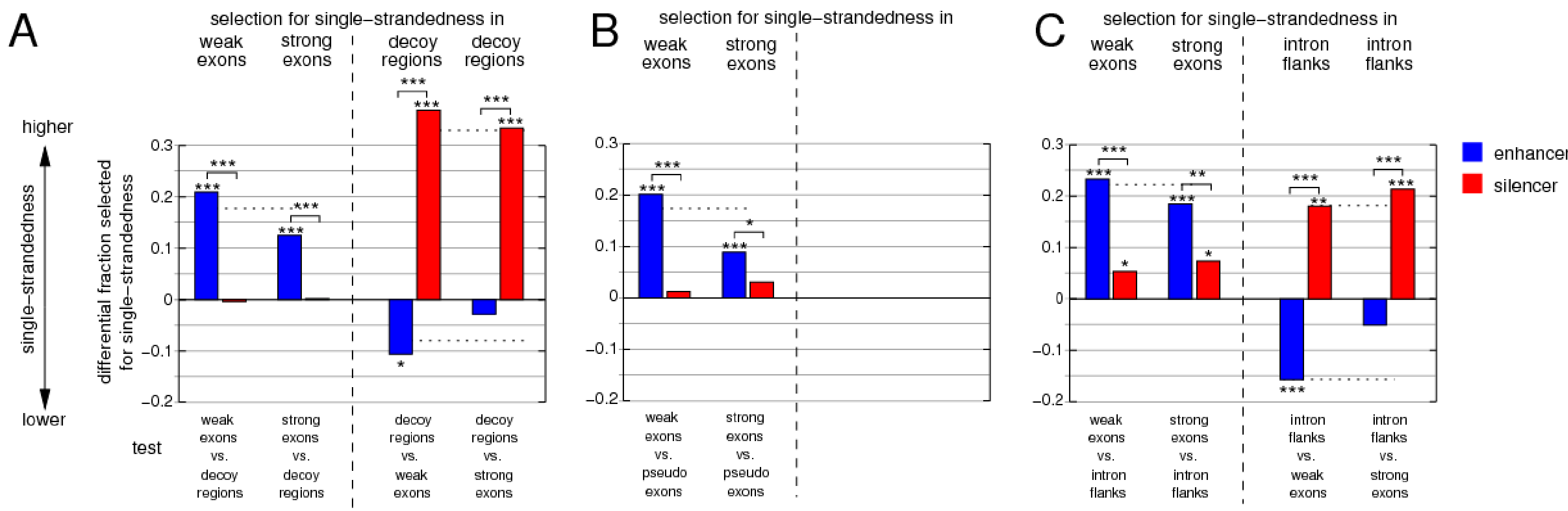

A
selection for single–strandedness in
weak exons
strong exons
decoy regions
decoy regions
higher
lower
single–strandedness
differential fraction selected for single–strandedness
0.3
0.2
0.1
0
–0.1
–0.2
***
**
*
test
weak exons vs. decoy regions
strong exons vs. decoy regions
decoy regions vs. weak exons
decoy regions vs. strong exons
B
selection for single–strandedness in
weak exons
strong exons
weak exons vs. pseudo exons
strong exons vs. pseudo exons
C
selection for single–strandedness in
weak exons
strong exons
intron flanks
intron flanks
weak exons vs. intron flanks
strong exons vs. intron flanks
intron flanks vs. weak exons
intron flanks vs. strong exons
enhancer
silencer
