# Supplementary material for: Pre-mRNA Secondary Structures Influence Exon Recognition
Source: PLoS Genet. 2007 Nov 16;3(11):e204. doi: 10.1371/journal.pgen.0030204 (PMC2077896; doi:10.1371/journal.pgen.0030204)
Supplement: Supplementary file 6 [file pgen.0030204.sg005.pdf]

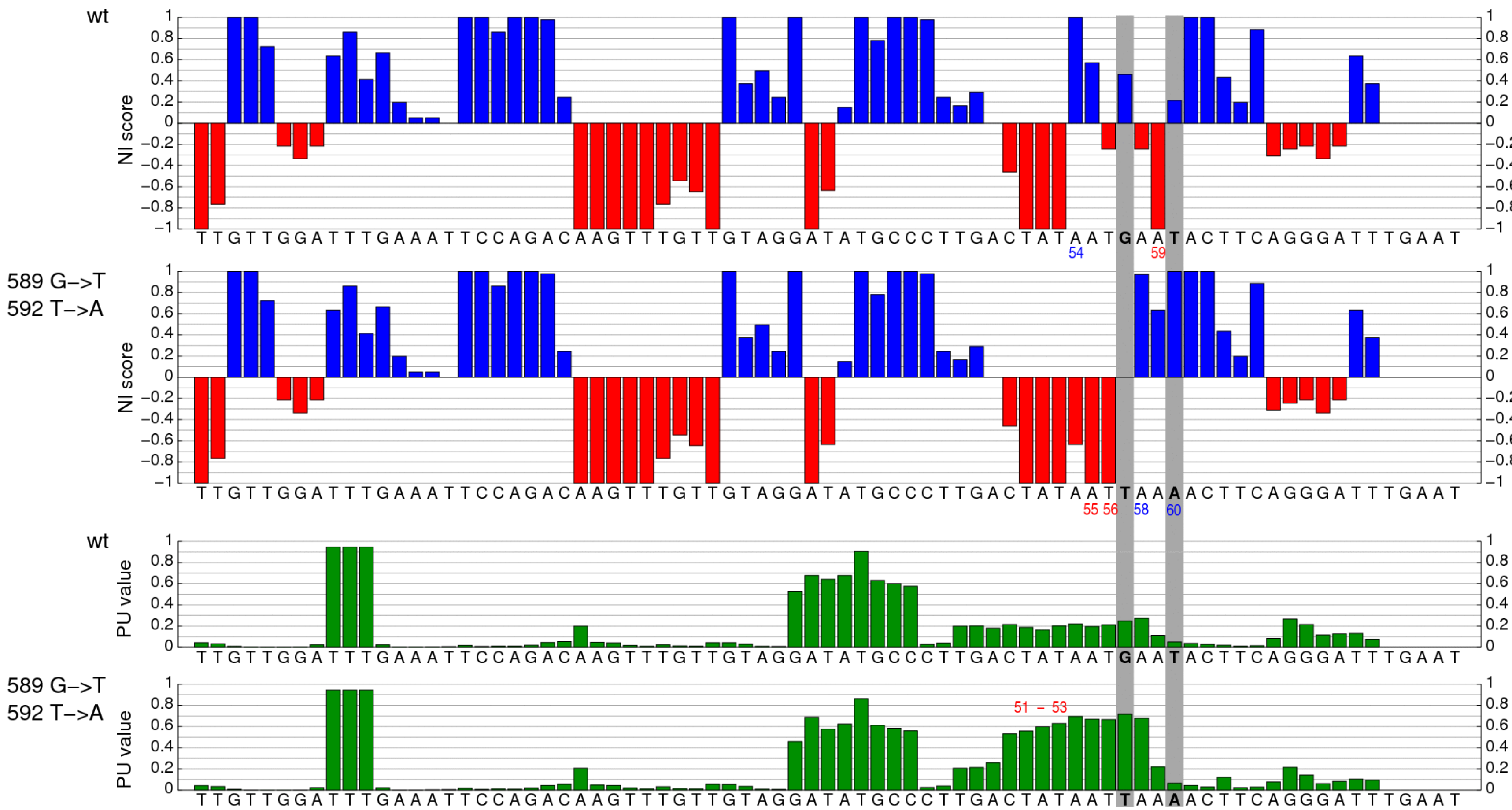
wt
589 G->T
592 T->A
wt
589 G->T
592 T->A
NI score
PU value
54
59
55 56
58
60
51 – 53
TTGTTGGATTTGAAATTCCAGACAAGTTTGTTGTAGGATATGCCCTTGACTATAATGAATACTTCAGGGATTTGAAT
TTGTTGGATTTGAAATTCCAGACAAGTTTGTTGTAGGATATGCCCTTGACTATAATTAAAACTTCAGGGATTTGAAT
