# Supplementary material for: Pre-mRNA Secondary Structures Influence Exon Recognition
Source: PLoS Genet. 2007 Nov 16;3(11):e204. doi: 10.1371/journal.pgen.0030204 (PMC2077896; doi:10.1371/journal.pgen.0030204)
Supplement: Supplementary file 7 [file pgen.0030204.sg006.pdf]

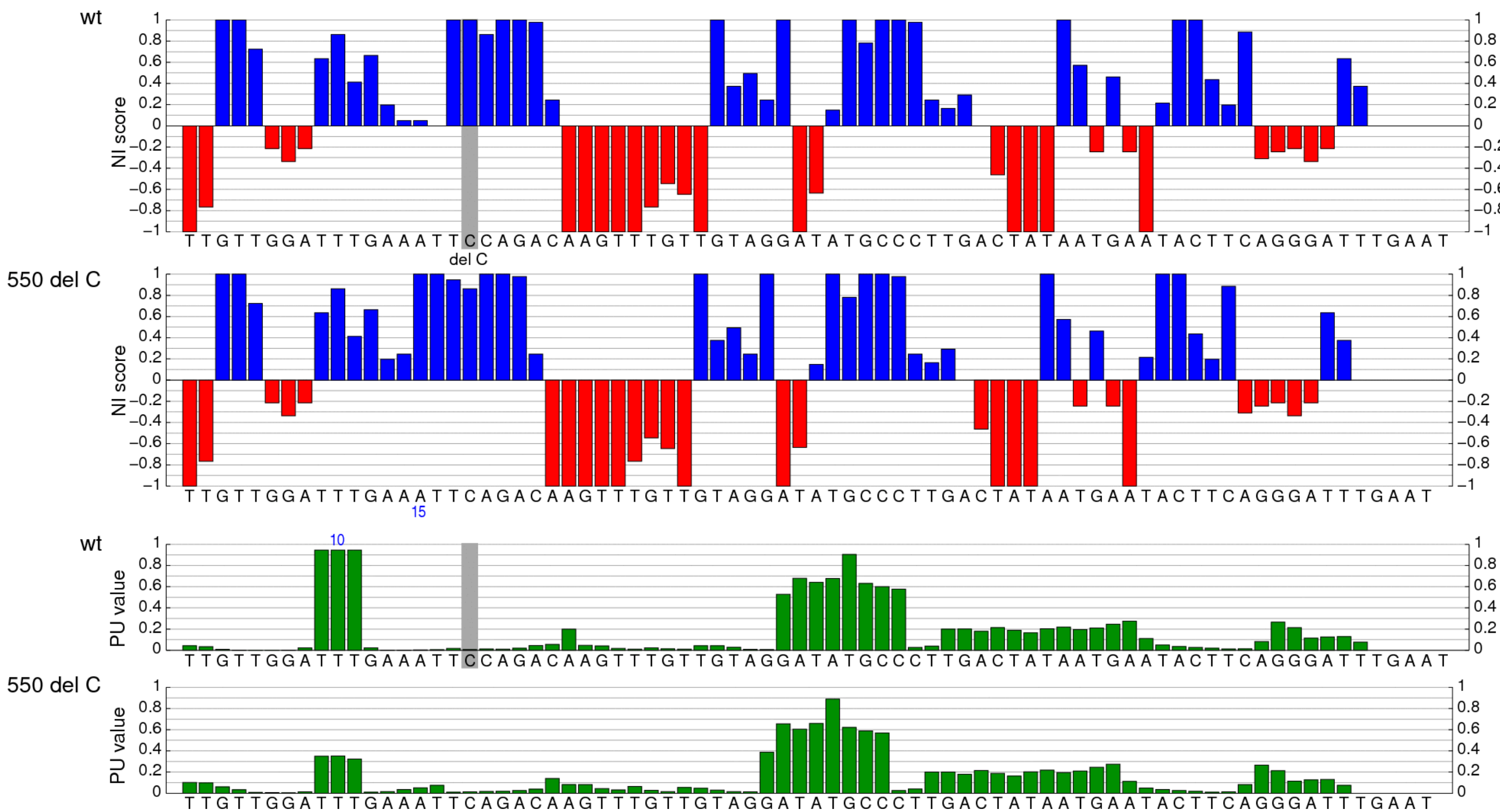

wt
NI score
T T G T T G G A T T T G A A A T T C C A G A C A A G T T T G T T G T A G G A T A T G C C C T T G A C T A T A A T G A A T A C T T C A G G G A T T T G A A T
del C
550 del C
NI score
T T G T T G G A T T T G A A A T T C A G A C A A G T T T G T T G T A G G A T A T G C C C T T G A C T A T A A T G A A T A C T T C A G G G A T T T G A A T
15
wt
10
PU value
T T G T T G G A T T T G A A A T T C C A G A C A A G T T T G T T G T A G G A T A T G C C C T T G A C T A T A A T G A A T A C T T C A G G G A T T T G A A T
550 del C
PU value
T T G T T G G A T T T G A A A T T C A G A C A A G T T T G T T G T A G G A T A T G C C C T T G A C T A T A A T G A A T A C T T C A G G G A T T T G A A T
