# Supplementary material for: Pre-mRNA Secondary Structures Influence Exon Recognition
Source: PLoS Genet. 2007 Nov 16;3(11):e204. doi: 10.1371/journal.pgen.0030204 (PMC2077896; doi:10.1371/journal.pgen.0030204)
Supplement: Supplementary file 8 [file pgen.0030204.sg007.pdf]

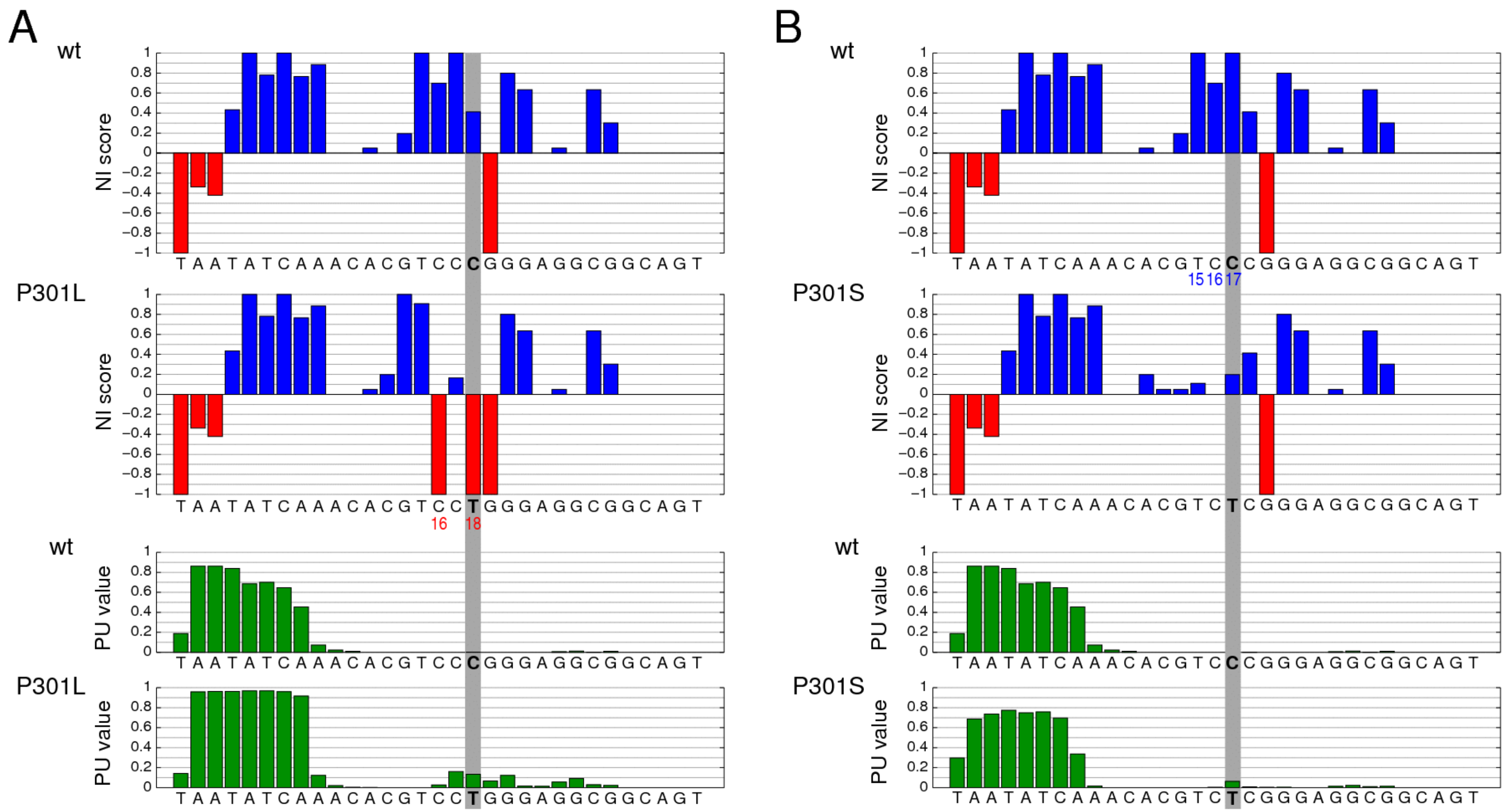

A
B
wt
P301L
P301S
NI score
PU value
T A A T A T C A A A C A C G T C C C G G G A G G C G G C A G T
T A A T A T C A A A C A C G T C C T G G G A G G C G G C A G T
T A A T A T C A A A C A C G T C C C G G G A G G C G G C A G T
T A A T A T C A A A C A C G T C T C G G G A G G C G G C A G T
16
18
15
16
17
