# Supplementary material for: Pre-mRNA Secondary Structures Influence Exon Recognition
Source: PLoS Genet. 2007 Nov 16;3(11):e204. doi: 10.1371/journal.pgen.0030204 (PMC2077896; doi:10.1371/journal.pgen.0030204)
Supplement: Supplementary file 9 [file pgen.0030204.sg008.pdf]

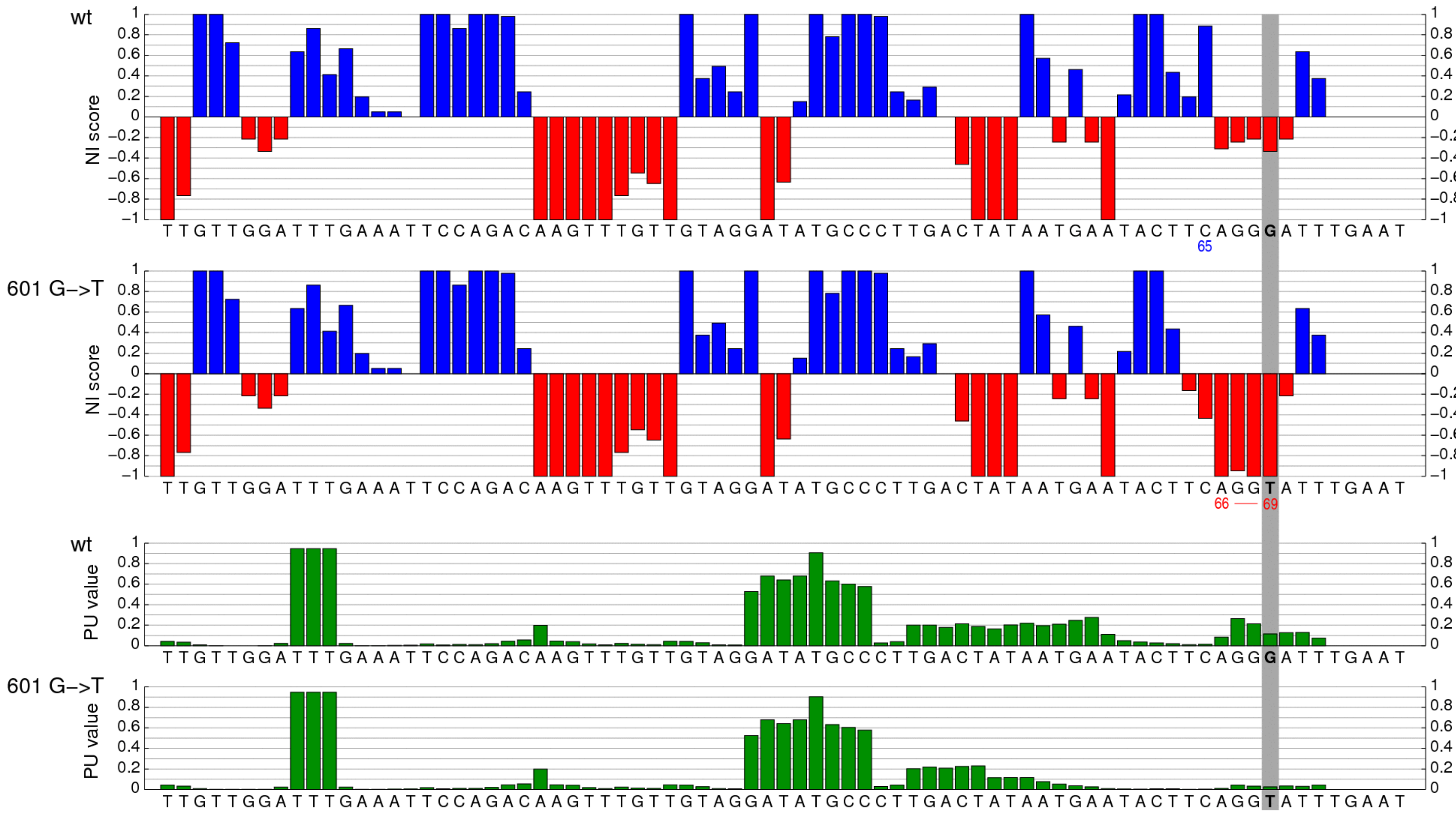
wt
NI score
1
0.8
0.6
0.4
0.2
0
−0.2
−0.4
−0.6
−0.8
−1
T T G T T G G A T T T G A A A T T C C A G A C A A G T T T G T T T G T A G G A T A T G C C C T T G A C T A T A A T G A A T A C T T C A G G G A T T T G A A T
65
601 G−>T
NI score
T T G T T G G A T T T G A A A T T C C A G A C A A G T T T G T T T G T A G G A T A T G C C C T T G A C T A T A A T G A A T A C T T C A G G T A T T T G A A T
66
69
wt
PU value
T T G T T G G A T T T G A A A T T C C A G A C A A G T T T G T T T G T A G G A T A T G C C C T T G A C T A T A A T G A A T A C T T C A G G G A T T T G A A T
601 G−>T
PU value
T T G T T G G A T T T G A A A T T C C A G A C A A G T T T G T T T G T A G G A T A T G C C C T T G A C T A T A A T G A A T A C T T C A G G T A T T T G A A T
