# Supplementary material for: Pre-mRNA Secondary Structures Influence Exon Recognition
Source: PLoS Genet. 2007 Nov 16;3(11):e204. doi: 10.1371/journal.pgen.0030204 (PMC2077896; doi:10.1371/journal.pgen.0030204)
Supplement: Supplementary file 11 [file pgen.0030204.st001.pdf]

Table S1

information about splicing motif								GC content motif				measurements of single-strandedness								
motif number	AEDB entry [a]	species	enhancer / silencer	exonic / intronic	gene name	motif	length of motif	motif	upstream 30 nt context	downstream 30 nt context	upstream context + motif + downstream context	EF20	PU20	ED20	EF30	PU30	ED30	EF50	PU50	ED50
1	db001	Homo_sapiens	ESE	exonic	CFTR	GATGAC	6	0.5	0.233	0.367	0.318	0.675	0.381	0.686	0.573	0.244	1.137	0.507	0.149	1.524
2	db004	Homo_sapiens	ESS	exonic	FN1	CAAGG	5	0.6	0.533	0.567	0.554	0.507	0.004	3.625	0.513	0.004	3.669	0.421	0.005	4.172
3	db005	Gallus_gallus	ESS	exonic	FINC	CATGG	5	0.6	0.6	0.5	0.554	0.379	0.003	3.623	0.328	0.002	3.858	0.3	0.002	4.057
4	db006	Homo_sapiens	ESE	exonic	FN1	GAAGAAGA	8	0.375	0.5	0.667	0.559	0.821	0.016	2.59	0.75	0.011	2.869	0.741	0.01	2.893
5	db007	Homo_sapiens	ESS	exonic	FN1	AAGTTTCC	8	0.375	0.533	0.6	0.544	0.571	0.124	2.78	0.479	0.067	3.537	0.469	0.034	4.356
6	db008	Gallus_gallus	ESE	exonic	FINC	GAAGAAGAC	9	0.444	0.633	0.633	0.609	0.774	0.047	2.029	0.737	0.031	2.312	0.7	0.021	2.551
7	db019	Homo_sapiens	ISE	intronic	GH1	GGGGATGGG	9	0.778	0.6	0.7	0.667	0.218	5.24E-007	9.295	0.2	3.00E-007	10.375	0.171	1.53E-007	11.363
8	db038	Human immunodeficiency virus 1	ESE	exonic	tat	GAAGAAGAA	9	0.333	0.667	0.5	0.551	0.983	0.856	0.097	0.986	0.881	0.08	0.904	0.598	0.55
9	db040	Human immunodeficiency virus 1	ESS	exonic	tat	TTGGGT	6	0.5	0.367	0.5	0.439	0.26	0.018	4.114	0.219	0.009	5.246	0.236	0.005	5.658
10	db042	Human immunodeficiency virus 1	ESS	exonic	env	GAAAGGAGA	9	0.444	0.4	0.333	0.377	0.718	0.304	1.639	0.551	0.156	2.681	0.487	0.078	4.151
11	db043	Human immunodeficiency virus 1	ESS	exonic	tat	AGATCC	6	0.5	0.5	0.4	0.455	0.52	0.032	2.59	0.406	0.016	3.678	0.319	0.009	3.916
12	db044	Human immunodeficiency virus 1	ESS	exonic	tat	TTAG	4	0.25	0.5	0.5	0.484	0.999	0.998	0.001	0.998	0.996	0.002	0.985	0.974	0.016
13	db049	Rattus_norvegicus	ESE	exonic	beta-TM	GAGGAGGAG	9	0.667	0.567	0.5	0.551	0.422	1.72E-006	8.379	0.398	5.30E-006	7.959	0.353	6.40E-006	7.677
14	db049	Rattus_norvegicus	ESE	exonic	beta-TM	GAGGAGGAG	9	0.667	0.8	0.567	0.681	0.588	0.001	4.743	0.457	0	6.09	0.358	0	6.687
15	db052	Rattus_norvegicus	ESS	exonic	beta-TM	TGTGGG	6	0.667	0.6	0.467	0.545	0.185	3.59E-006	8.227	0.178	2.00E-006	8.909	0.177	1.02E-006	9.739
16	db053	Rattus_norvegicus	ESE	exonic	beta-TM	GAAGAAGAG	9	0.444	0.333	0.467	0.406	0.538	0.031	2.36	0.554	0.056	1.991	0.525	0.037	2.442
17	db058	Homo_sapiens	ESS	exonic	FGFR2	TAGG	4	0.5	0.3	0.633	0.469	0.685	0.058	2.261	0.38	0.029	4.161	0.362	0.017	4.378
18	db065	Homo_sapiens	ESE	exonic	CD44	CAACCACAA	9	0.444	0.467	0.4	0.435	0.618	0.003	3.629	0.623	0.009	3.403	0.729	0.08	2.292
19	db075	Homo_sapiens	ESE	exonic	PTPRC	AACACCT	7	0.429	0.633	0.5	0.552	0.753	0.143	1.265	0.763	0.139	1.294	0.799	0.284	0.94
20	db078	Homo_sapiens	ESS	exonic	PTPRC	ACTCGCA	7	0.571	0.633	0.633	0.627	0.583	0.004	3.528	0.538	0.003	3.71	0.507	0.002	3.751
21	db079	Homo_sapiens	ESE	exonic	CALCA	TTTTTCCCC	9	0.444	0.267	0.467	0.377	0.378	0.037	4.291	0.357	0.018	5.527	0.398	0.033	4.128
22	db083	Rous sarcoma virus	ESS	exonic	gag	GGGG	4	1	0.533	0.533	0.563	0.781	0.324	0.81	0.783	0.341	0.735	0.767	0.327	0.776
23	db083	Rous sarcoma virus	ESS	exonic	gag	GGG	3	1	0.6	0.467	0.556	0.806	0.41	0.556	0.825	0.473	0.47	0.801	0.422	0.543
24	db085	Human adenovirus type 2	ESE	exonic	E1A	GACGACGAG	9	0.667	0.633	0.433	0.551	0.805	0.229	1.081	0.848	0.335	0.792	0.844	0.339	0.732
25	db087	Human adenovirus type 2	ESE	exonic	E1A	GATGAAGAG	9	0.444	0.633	0.433	0.522	0.852	0.258	1.496	0.821	0.136	2.058	0.807	0.075	2.343
26	db089	Homo_sapiens	ESE	exonic	BRCA1	GCTGAGT	7	0.571	0.333	0.367	0.373	0.669	0.007	3.382	0.694	0.004	3.563	0.662	0.003	3.694
27	db091	Homo_sapiens	ESE	exonic	MAPT	AATAAGAAG	9	0.222	0.367	0.5	0.406	0.82	0.216	0.971	0.85	0.166	1.296	0.814	0.103	1.639
28	db101	Homo_sapiens	ESS	exonic	ATP5C1	AGTTCCA	7	0.429	0.233	0.4	0.328	0.573	0.348	2.007	0.391	0.176	2.811	0.46	0.221	2.038
29	db102	Mice minute virus	ESE	exonic	NS2	GAAGAAAGA	9	0.333	0.567	0.567	0.536	0.584	1.25E-005	7.543	0.572	0	6.323	0.57	0	5.723
30	db103	Mice minute virus	ESE	exonic	NS2	CACCA	5	0.6	0.433	0.533	0.492	0.917	0.766	0.173	0.926	0.786	0.152	0.862	0.596	0.427
31	db103	Mice minute virus	ESE	exonic	NS2	ACACC	5	0.6	0.533	0.5	0.523	0.924	0.746	0.191	0.919	0.721	0.208	0.905	0.679	0.262

32	db107	Homo_sapiens	ESE	exonic	SMN1	AAAGAAGG	8	0.375	0.333	0.367	0.353	0.758	0.184	2.541	0.638	0.092	3.715	0.488	0.046	4.756
33	db109	Homo_sapiens	ESS	exonic	SMN1	TAGACA	6	0.333	0.3	0.367	0.333	0.809	0.286	0.773	0.774	0.247	0.886	0.707	0.184	1.106
34	db110	Homo_sapiens	ESE	exonic	SFRS7	GAAGAAGAA	9	0.333	0.467	0.267	0.362	0.907	0.786	0.171	0.784	0.58	0.541	0.679	0.415	0.814
35	db116	Gallus_gallus	ESS	exonic	MYPT1	AACACAAG	8	0.375	0.367	0.367	0.368	0.732	0.312	0.808	0.764	0.357	0.684	0.772	0.38	0.622
36	db117	Mus_musculus	ESS	exonic	Epb4.1	ATAGCA	6	0.333	0.333	0.3	0.318	0.537	0.02	2.623	0.451	0.01	3.788	0.384	0.006	4.142
37	db123	Homo_sapiens	ESE	exonic	TPM3	AAGTGTT	7	0.286	0.333	0.533	0.418	0.757	0.093	1.492	0.722	0.085	1.871	0.693	0.079	1.772
38	db125	Gallus_gallus	ESE	exonic	TRT2	GAGGAAGAA	9	0.444	0.533	0.533	0.522	0.816	0.603	0.328	0.669	0.386	1.061	0.422	0.193	4.34
39	db131	Homo_sapiens	ESS	exonic	CFTR	GGGGG	5	1	0.3	0.567	0.477	0.677	0.363	0.726	0.591	0.277	0.914	0.627	0.253	0.919
40	db151	Mus_musculus	ISE	intronic	Agrn	ATCTTTTGT	9	0.222	0.5	0.5	0.464	0.769	0.246	0.995	0.76	0.265	0.917	0.778	0.329	0.761
41	db152	Mus_musculus	ISE	intronic	Agrn	ACTCACC	7	0.571	0.467	0.433	0.463	0.914	0.713	0.209	0.738	0.395	1.014	0.654	0.259	1.216
42	db152	Mus_musculus	ISE	intronic	Agrn	TTACC	5	0.4	0.5	0.4	0.446	0.993	0.978	0.013	0.993	0.978	0.014	0.976	0.904	0.078
43	db155	Homo_sapiens	ISE	intronic	APP	ATGTTT	6	0.167	0.533	0.233	0.364	0.877	0.716	0.208	0.812	0.57	0.383	0.773	0.46	0.529
44	db156	Homo_sapiens	ISS	intronic	APP	TTT	3	0	0.433	0.2	0.302	0.985	0.957	0.027	0.981	0.945	0.035	0.975	0.929	0.045
45	db164	Homo_sapiens	ESE	exonic	TRA2B	GAAAGAA	7	0.286	0.2	0.433	0.313	0.895	0.661	0.27	0.838	0.438	0.609	0.818	0.364	0.685
46	db164	Homo_sapiens	ESE	exonic	TRA2B	GAAAGAAG	8	0.375	0.4	0.3	0.353	0.838	0.108	1.41	0.855	0.111	1.372	0.858	0.166	1.164
47	db164	Homo_sapiens	ESE	exonic	TRA2B	GAAGAAGG	8	0.5	0.267	0.367	0.338	0.778	0.544	0.721	0.422	0.273	3.234	0.284	0.136	5.136
48	db167	Mus_musculus	ISS	intronic	Casp2	TTTCTCTC	8	0.375	0.4	0.6	0.485	0.997	0.976	0.015	0.997	0.974	0.016	0.996	0.967	0.02
49	db167	Mus_musculus	ISS	intronic	Casp2	TCTTC	5	0.4	0.367	0.367	0.369	0.871	0.651	0.349	0.868	0.505	0.491	0.867	0.434	0.559
50	db170	Mus_musculus	ISS	intronic	Src	TCTCTCT	7	0.429	0.567	0.6	0.567	0.96	0.689	0.237	0.95	0.604	0.321	0.929	0.529	0.406
51	db170	Mus_musculus	ISS	intronic	Src	CTCTCT	6	0.5	0.633	0.6	0.606	0.981	0.9	0.066	0.927	0.707	0.375	0.774	0.374	1.381
52	db171	Mus_musculus	ISS	intronic	Src	CTCTC	5	0.6	0.7	0.633	0.662	0.714	0.009	3.024	0.701	0.008	3.188	0.681	0.007	3.315
53	db171	Mus_musculus	ISE	intronic	Src	TGCATG	6	0.5	0.667	0.6	0.621	0.803	0.128	1.278	0.809	0.131	1.259	0.825	0.136	1.234
54	db171	Mus_musculus	ISE	intronic	Src	GGGGGCTG	8	0.875	0.633	0.633	0.662	0.625	0.002	4.081	0.665	0.002	3.961	0.577	0.001	4.223
55	db174	Homo_sapiens	ISS	intronic	FGFR1	TGCTGC	6	0.667	0.433	0.433	0.455	0.377	0.028	2.294	0.389	0.02	2.576	0.385	0.018	2.603
56	db174	Homo_sapiens	ISS	intronic	FGFR1	TTGCCTCT	8	0.5	0.467	0.467	0.471	0.835	0.454	0.514	0.802	0.421	0.942	0.597	0.21	4.312
57	db174	Homo_sapiens	ISS	intronic	FGFR1	CTGCTTCC	8	0.625	0.433	0.333	0.412	0.681	0.01	3.213	0.681	0.014	2.917	0.596	0.016	3.032
58	db175	Homo_sapiens	ISS	intronic	FGFR1	TGCTGC	6	0.667	0.567	0.633	0.606	0.444	0.09	1.662	0.478	0.095	1.545	0.51	0.068	1.762
59	db175	Homo_sapiens	ISS	intronic	FGFR1	TGCCTGC	7	0.714	0.533	0.6	0.582	0.37	0.002	4.665	0.349	0.001	4.739	0.331	0.001	5.38
60	db187	Rattus_norvegicus	ISE	intronic	GBRG2	ACAAATCCA	9	0.333	0.3	0.4	0.348	0.805	0.318	0.926	0.806	0.233	1.056	0.744	0.144	1.507
61	db190	Mus_musculus	ISS	intronic	P70370	TAGAGT	6	0.333	0.367	0.367	0.364	0.684	0.314	0.846	0.683	0.206	1.138	0.681	0.15	1.296
62	db196	Bovine papillomavirus - 1	ESS	exonic	late mRNA	TCTTCTT	7	0.286	0.633	0.733	0.642	0.876	0.33	0.728	0.881	0.483	0.503	0.882	0.559	0.392
63	db198	Bovine papillomavirus - 1	ESS	exonic	late mRNA	GGCTCCCCC	9	0.889	0.6	0.7	0.681	0.667	0	5.682	0.658	9.98E-005	5.775	0.635	0	5.798
64	db200	Bovine papillomavirus - 1	ESS	exonic	late mRNA	AGAGCAGG	8	0.625	0.733	0.7	0.706	0.446	0	5.281	0.422	0	5.429	0.313	0	6.484
65	db202	Bovine papillomavirus - 1	ESS	exonic	late mRNA	TGGT	4	0.5	0.567	0.5	0.531	0.183	0.002	4.389	0.242	0.001	5.048	0.255	0.001	5.174
66	db206	Bovine papillomavirus - 1	ESE	exonic	late mRNA	GAAGAA	6	0.333	0.633	0.6	0.591	0.683	0.21	1.09	0.647	0.129	1.507	0.647	0.082	1.79
67	db206	Bovine papillomavirus - 1	ESE	exonic	late mRNA	GAAGAA	6	0.333	0.6	0.633	0.591	0.882	0.409	0.889	0.858	0.244	1.226	0.837	0.156	1.449

68	db212	Mus_musculus	ISS	intronic	Kcnma1	CTCTTCCTC	9	0.556	0.5	0.5	0.507	0.973	0.815	0.132	0.947	0.683	0.25	0.921	0.628	0.427
69	db214	Homo_sapiens	ISS	intronic	MAPT	TCACACGT	8	0.5	0.667	0.533	0.588	0.132	0	5.462	0.112	0.001	5.016	0.1	0	5.206
70	db215	Homo_sapiens	ISS	intronic	MAPT	CCCATGCG	8	0.75	0.6	0.467	0.559	0.583	0.085	2.069	0.574	0.044	2.943	0.537	0.031	3.787
71	db216	Rattus_norvegicus	ISE	intronic	THA	GAGGGAG	7	0.714	0.6	0.567	0.597	0.899	0.618	0.297	0.892	0.61	0.305	0.89	0.605	0.31
72	db216	Rattus_norvegicus	ISE	intronic	THA	GGGTG	5	0.8	0.567	0.667	0.631	0.713	0.199	1.057	0.769	0.194	1.043	0.763	0.183	1.065
73	db219	Rattus_norvegicus	ISS	intronic	alpha-TM	TCTTCTT	7	0.286	0.367	0.6	0.463	0.999	0.991	0.006	0.975	0.85	0.149	0.784	0.477	1.905
74	db231	Gallus_gallus	ISE	intronic	MYPT1	TCTTGTCTT	9	0.333	0.367	0.267	0.319	0.442	0.013	3.396	0.379	0.007	5.918	0.379	0.006	4.957
75	db239	Homo_sapiens	ESE	exonic	CFTR	GGATAC	6	0.5	0.3	0.2	0.273	0.533	0.012	2.811	0.353	0.007	3.466	0.337	0.005	3.535
76	db239	Homo_sapiens	ESE	exonic	CFTR	AAGATGC	7	0.429	0.267	0.3	0.299	0.453	0	4.808	0.426	0.001	4.569	0.456	0.001	4.64
77	db284	Mus_musculus	ESE	exonic	ATP5C1	AATGAAAA	8	0.125	0.267	0.433	0.324	0.655	0.029	2.256	0.663	0.075	1.922	0.578	0.054	2.03

[a] identifier for each AEDB motif database entry; please note that the literature references in one entry can describe more than one splicing motif
