# Supplementary material for: Pre-mRNA Secondary Structures Influence Exon Recognition
Source: PLoS Genet. 2007 Nov 16;3(11):e204. doi: 10.1371/journal.pgen.0030204 (PMC2077896; doi:10.1371/journal.pgen.0030204)
Supplement: Supplementary file 12 [file pgen.0030204.st002.pdf]

	number of motifs	average PU						average EF						average ED					
		**11...20 nt**		**11...30 nt**		**11...50 nt**		**11...20 nt**		**11...30 nt**		**11...50 nt**		**11...20 nt**		**11...30 nt**		**11...50 nt**	
splicing motifs	77	0.29		0.25		0.21		0.69		0.65		0.62		2.17		2.49		2.77	
exonic splicing motifs	50	0.23		0.19		0.16		0.67		0.62		0.59		2.41		2.77		3.02	
intronic splicing motifs	27	0.40		0.35		0.29		0.73		0.71		0.67		1.72		1.96		2.32	
control 1	100 x 77	0.18	P<0.01	0.15	P<0.01	0.13	P<0.01	0.63	P<0.01	0.59	P<0.01	0.55	P<0.01	2.59	P<0.03	2.86	P<0.04	3.16	P<0.02
control 2	100 x 77	0.21	P<0.01	0.18	P<0.01	0.15	P<0.01	0.64	P<0.02	0.60	P<0.01	0.57	P<0.01	2.60	P<0.04	2.91	P<0.05	3.19	P<0.04
control 3	100 x 77	0.18	P<0.01	0.15	P<0.01	0.12	P<0.01	0.63	P<0.01	0.59	P<0.01	0.55	P<0.01	2.61	P<0.01	2.91	P<0.01	3.22	P<0.02
control 4	10000	0.14	P<0.020	0.12	P<0.023	0.09	P<0.018	0.60	P<0.005	0.57	P<0.047	0.53	P<0.046	2.83	*P=0.054*	3.12	*P=0.154*	3.40	*P=0.134*
control 4 – lower GC content	10000	0.15	*P=0.051*	0.12	*P=0.064*	0.10	*P=0.051*	0.61	P<0.013	0.58	*P=0.100*	0.54	*P=0.097*	2.67	*P=0.140*	2.95	*P=0.348*	3.22	*P=0.316*
control 4 – equal GC content	10000	0.15	P<0.041	0.12	P<0.046	0.10	P<0.034	0.61	P<0.010	0.57	*P=0.082*	0.53	*P=0.069*	2.73	*P=0.105*	3.02	*P=0.267*	3.31	*P=0.220*
control 4 – higher GC content	10000	0.12	P<0.002	0.10	P<0.002	0.08	P<0.002	0.59	P<0.001	0.55	P<0.013	0.51	P<0.012	3.07	P<0.006	3.37	P<0.022	3.68	P<0.017
control 5	10000	0.18	P<0.004	0.15	P<0.003	0.12	P<0.003	0.62	P<0.004	0.58	P<0.001	0.54	P<0.001	2.52	P<0.003	2.83	P<0.002	3.14	P<0.007
control 5 – higher GC content	10000	0.13	P<0.001	0.11	P<0.001	0.08	P<0.001	0.58	P<0.001	0.54	P<0.001	0.50	P<0.001	3.26	P<0.001	3.60	P<0.001	3.97	P<0.001

Table S2: PU, EF, ED values, and P-values for verified splicing motifs and all controls.
