# Supplementary material for: Pre-mRNA Secondary Structures Influence Exon Recognition
Source: PLoS Genet. 2007 Nov 16;3(11):e204. doi: 10.1371/journal.pgen.0030204 (PMC2077896; doi:10.1371/journal.pgen.0030204)
Supplement: Supplementary file 13 [file pgen.0030204.st003.pdf]

	number of motifs	average GC content		
		motif	30 nt upstream	30 nt downstream
splicing motifs	77	0.49	0.48	0.49
exonic splicing motifs	50	0.49	0.47	0.48
intronic splicing motifs	27	0.50	0.51	0.49
control 1	100 x 77	0.48	0.48	0.49
control 2	100 x 77	0.49	**0.49**	0.49
control 3	100 x 77	0.49	0.48	0.48
control 4	10000	**0.50**	**0.50**	**0.50**
control 4 – lower GC content	10000	0.47	0.46	0.47
control 4 – equal GC content	10000	0.49	0.47	0.48
control 4 – higher GC content	10000	**0.57**	**0.53**	**0.53**
control 5	10000	0.43	0.43	0.43
control 5 – higher GC content	10000	**0.60**	**0.55**	**0.55**

Table S3: GC content for the verified motif data sets and all controls.
