# Supplementary material for: Pre-mRNA Secondary Structures Influence Exon Recognition
Source: PLoS Genet. 2007 Nov 16;3(11):e204. doi: 10.1371/journal.pgen.0030204 (PMC2077896; doi:10.1371/journal.pgen.0030204)
Supplement: Supplementary file 14 [file pgen.0030204.st004.pdf]

exons vs. decoy regions

selection for single-strandedness in exons:

773 of 2268 neutral motifs	34.08%			
450 of 867 ESE motifs	51.90%		differential fraction	17.82%
149 of 457 ESS motifs	32.60%		differential fraction	-1.48%
Fisher´s exact test:		ESE vs. ESS	P-value: <0.0001	(odds ratio 2.23)
		ESE vs. neutral	P-value: <0.0001	(odds ratio 2.09)
		ESS vs. neutral	P-value: 0.5517	(odds ratio 1.07)

selection for single-strandedness in decoy regions:

211 of 353 neutral motifs	59.77%			
58 of 112 ESE motifs	51.79%		differential fraction	-7.99%
36 of 39 ESS motifs	92.31%		differential fraction	32.53%
Fisher´s exact test:		ESE vs. ESS	P-value: <0.0001	(odds ratio 11.17)
		ESE vs. neutral	P-value: 0.1536	(odds ratio 1.38)
		ESS vs. neutral	P-value: <0.0001	(odds ratio 8.08)

---

exons vs. pseudo exons

selection for single-strandedness in exons:

684 of 2618 neutral motifs	26.13%			
426 of 979 ESE motifs	43.51%		differential fraction	17.39%
133 of 496 ESS motifs	26.81%		differential fraction	0.69%
Fisher´s exact test:		ESE vs. ESS	P-value: <0.0001	(odds ratio 2.10)
		ESE vs. neutral	P-value: <0.0001	(odds ratio 2.18)
		ESS vs. neutral	P-value: 0.7388	(odds ratio 0.97)

selection for single-strandedness in pseudo exons:

2 of 3 neutral motifs	66.67%			
0 of 0 ESE motifs	0.00%		differential fraction	-
0 of 0 ESS motifs	0.00%		differential fraction	-

---

exons vs. intron flanks

selection for single-strandedness in exons:

515 of 1820 neutral motifs	28.30%			
343 of 675 ESE motifs	50.81%		differential fraction	22.52%
145 of 439 ESS motifs	33.03%		differential fraction	4.73%
Fisher´s exact test:		ESE vs. ESS	P-value: <0.0001	(odds ratio 2.09)
		ESE vs. neutral	P-value: <0.0001	(odds ratio 2.62)
		ESS vs. neutral	P-value: 0.0537	(odds ratio 0.80)

selection for single-strandedness in intron flanks:

583 of 801 neutral motifs	72.78%			
173 of 304 ESE motifs	56.91%		differential fraction	-15.88%
52 of 57 ESS motifs	91.23%		differential fraction	18.44%
Fisher´s exact test:		ESE vs. ESS	P-value: <0.0001	(odds ratio 7.88)
		ESE vs. neutral	P-value: <0.0001	(odds ratio 2.03)

Table S4: Number of ESEs, ESSs and neutral motifs that are selected for single-strandedness comparing exons with decoy regions, pseudo exons and intron flanks.
