# Supplementary material for: Pre-mRNA Secondary Structures Influence Exon Recognition
Source: PLoS Genet. 2007 Nov 16;3(11):e204. doi: 10.1371/journal.pgen.0030204 (PMC2077896; doi:10.1371/journal.pgen.0030204)
Supplement: Supplementary file 15 [file pgen.0030204.st005.pdf]

	#winners [a]	total [b]	%	Fisher´s exact test		% difference	Fisher´s exact test		% difference
**exons vs. pseudo exons**									
ESE	478	979	48.8%	-	-	-			
ESS	194	496	39.1%	ESE vs. ESS	P<0.001	9.7%			
Neutral	1118	2621	42.7%	ESE vs. neutral	P=0.001	6.1%	ESS vs. neutral	*P=0.15*	-3.6%
**exons vs. decoy regions**									
ESE	513	979	52.4%	-	-	-			
ESS	193	496	38.9%	ESE vs. ESS	P<0.001	13.5%			
Neutral	1198	2621	45.7%	ESE vs. neutral	P<0.001	6.7%	ESS vs. neutral	P=0.006	-6.8%
**exons vs. intron flanks**									
ESE	489	979	49.9%	-	-	-			
ESS	202	496	40.7%	ESE vs. ESS	P<0.001	9.2%			
Neutral	1119	2621	42.7%	ESE vs. neutral	P<0.001	7.2%	ESS vs. neutral	*P=0.43*	-2.0%

Table S5: Comparison of PU values of splicing motifs between data sets.
