# Supplementary material for: Pre-mRNA Secondary Structures Influence Exon Recognition
Source: PLoS Genet. 2007 Nov 16;3(11):e204. doi: 10.1371/journal.pgen.0030204 (PMC2077896; doi:10.1371/journal.pgen.0030204)
Supplement: Supplementary file 16 [file pgen.0030204.st006.pdf]

	#winners [a]	total [b]	%	# winners [a]	total [b]	%	Fisher's exact test	% difference
		**within exons**			**within pseudo exons**			
ESE vs. Neutral	556	979	56.8%	494	979	50.5%	P = 0.0057	6.3%
ESS vs. Neutral	108	496	21.8%	156	496	31.5%	P = 0.0007	-9.7%
		**within exons**			**within decoy regions**			
ESE vs. Neutral	556	979	56.8%	465	979	47.5%	P < 0.0001	9.3%
ESS vs. Neutral	108	496	21.8%	191	496	38.5%	P < 0.0001	-16.7%
		**within exons**			**within intron flanks**			
ESE vs. Neutral	556	979	56.8%	471	979	48.1%	P = 0.0001	8.7%
ESS vs. Neutral	108	496	21.8%	165	496	33.3%	P < 0.0001	-11.5%

Table S6: Comparison of PU values within data sets.
